# Perceptions of sexual assault perpetrators, victims, and event depend on system justification beliefs and perpetrator atonement

**DOI:** 10.1371/journal.pone.0311983

**Published:** 2024-12-31

**Authors:** Brianna C. Delker, Kira K. Means, Allison Schwam, Aubrie L. Patterson, Camille A. Fogel, Amelita Brown, Alex M. Czopp, Kate C. McLean

**Affiliations:** Department of Psychology, Western Washington University, Bellingham, Washington, United States of America; Philipps-Universitat Marburg, GERMANY

## Abstract

When accused of wrongdoing, a sexual assault perpetrator may express atonement, i.e., he may acknowledge harm done, take responsibility, and make amends. Anecdotal observations suggest that mainstream U.S. audiences respond favorably when high-status perpetrators express *less* atonement, such as telling stories that minimize harm, or place responsibility on the victim. However, empirically, little is known about how perpetrator status and atonement influence audience responses. Informed by system justification theory, this vignette-based experiment tested the hypothesis that the more audiences are psychologically invested in an unequal status quo (i.e., the greater their system justification beliefs), the more they will favor perpetrators (vs. victims), *especially* when high-status perpetrators atone less, and low-status perpetrators atone more. In a pre-registered 2(*perpetrator status*: low, high) x 3(*perpetrator narrative atonement*: low, medium, high) x continuous(*participant system justification*) between-subjects design, U.S. adults (*N* = 895) were randomly assigned to read 1 of 6 first-person stories by a white male who has been accused of sexual assault by a female acquaintance. Dependent measures included perceived severity of and relative responsibility for the assault, empathy toward perpetrator and victim, and ratings of their likeability and positive personality traits. Hierarchical regression analysis revealed that, instead of the hypothesized interactive effects, there were consistent main effects of system justification and atonement across perpetrator status levels. The greater their system justification beliefs, the more participants favored perpetrators, the less severe they rated the assault, and the less they favored victims. Greater perpetrator atonement boosted favorability ratings for him *and* the victim. Conversely, *less* perpetrator atonement diminished his favorability ratings, but also made the assault appear less severe and less his (vs. the victim’s) responsibility. Findings underscore the strong influences that perpetrator stories and psychological investment in an unequal status quo have on perceptions of sexual violence.

## Introduction

Growing public awareness of how common sexual violence is can be attributed, in part, to the #MeToo movement and to survivors who have come forward to share their stories. Despite this public reckoning, prevalence rates of interpersonal violence remain stable [1] and cultural stigma surrounding victimization persists, characterized by patterns of disbelief, victim-blame, and well-received denials by the accused [2]. Whether assaults happen at high school parties in small towns, Ivy League university campuses, or the highest-ranking political offices, mainstream U.S. audiences apparently continue to reject credible survivor stories of assault in favor of denial of wrongdoing, especially when the accused is of high status (e.g., based on wealth, occupation, reputation). Research evidence for a bias favoring high-status perpetrators is more mixed, however: audiences have been found to perceive high-status male perpetrators as both more [3] and less [4] credible than low-status male perpetrators in vignette-based experiments. The purpose of the present study is to unpack the conditions under which audiences might favor the stories of high- versus low-status male perpetrators, at the potential expense of victims.

To better understand the story preferences of mainstream U.S. audiences, we took a structural-psychological approach which assumes that individual psychology is embedded within (and must reckon with) societal systems of power, oppression, and inequality [5]. Audience evaluations of sexual assault perpetrator stories and status occur in the setting of major status-quo inequalities in the U.S., including economic inequality [6], and gender-based inequalities in interpersonal violence. Most people who commit sexual assault are cisgender men, and most victims are cisgender women and transgender people [7, 8]. In the present study, we propose that the strength of psychological investment in the legitimacy of the status quo (i.e., *system justification*) will explain the conditions under which audiences favor stories of high- versus low-status perpetrators. Further, we propose that the type of story that a perpetrator tells—if he denies wrongdoing versus takes accountability and narratively expresses *atonement* for his actions—is an important feature in evaluations of his and the victim’s credibility, in ways unpacked further below.

### System justification theory defined and applied to sexual assault perpetrator status

According to system justification theory, people often hold a fundamental motivation to believe that extant differences in wealth, power, and status between groups are fair, legitimate, and inevitable [9]. One tenet of system justification theory posits that when faced with information that threatens the legitimacy of the status quo, people will revise, often unconsciously, their evaluations of perpetrators and victims to rationalize the status quo that they perceive to be fair. For instance, when the perceived fairness of the social system was threatened by the U.S. government’s failed response to Hurricane Katrina—a failure which disproportionately harmed people living in poverty and African Americans—audiences responded with derogating judgments of suffering New Orleans residents as criminal (“looting” stores for food) and aggressive [10]. Likewise, when social system threat is experimentally manipulated, people respond defensively with derogating judgments of powerless people (as less intelligent and independent) and enhancing judgments of powerful people (as more intelligent and independent) [11].

In the context of sexual assault, when a high-status man (e.g., wealthy, powerful) is accused of criminal behavior, this information threatens the notion that those in positions of power deserve and will remain in their superior economic and reputational position. However, when a high-status man responds to the accusation with a denial of wrongdoing and defense of his innocence, audiences with strong system-justifying tendencies can accept his denial and consequently be reassured that the (inequitable) economic status quo is legitimate. In this scenario, “blameless” high-status group members remain safely and predictably in their superior economic position, with victims predictably in their degraded position. By contrast, a low-status perpetrator’s denial would be likely to elicit more negative evaluations. For audiences who are particularly motivated to preserve the status quo, a low-status perpetrator’s denial of wrongdoing is inconsistent with system-justifying beliefs in a just world that designate low-status individuals as responsible for their lowly economic circumstances. Less clear is how a low-status perpetrator’s denial would shape audience evaluations of the *victim’s* credibility, a question that will be explored in this study.

### Perpetrator narrative atonement

A favorable response to denial of wrongdoing is, to some degree, at odds with the dominant U.S. cultural tendency to celebrate redemptive stories of going from bad to good, a low point to a place of triumphant personal growth and meaning made [12]. A specific form of redemption relevant to acts of transgression is atonement: when a transgressor atones, he acknowledges harm done and makes appropriate amends [13]. Atonement is a multidimensional construct that encompasses potential gradations in emotion (from self-focused emotions such as shame to more other-focused emotions such as guilt and compassion); apology (from relatively self-focused to other-focused); responsibility-taking; and positive behavior change [14, 15]. System justification theory predicts that audiences with strong motives to protect the status quo will respond favorably only when low-status (not high-status) perpetrators atone for wrongdoing. When the accused is already mired among the lower rungs of the economic ladder, audiences can potentially welcome his accountability-taking and apology without fear that these will alter his status, thus maintaining the status quo. Further, the atonement of a low-status perpetrator may serve a palliative function that makes it easier to accept and legitimize his inferior position in society [11, 16].

### Current study

Sexual assault is a private trauma that tends to occur behind closed doors. The way that others learn about a sexual assault and its personal consequences, then, is through the victim’s and/or perpetrator’s ability and willingness to provide a narrative of what happened. Public perceptions of sexual assault victims and perpetrators are therefore entangled with what type of stories they tell [17, 18], along with hegemonic cultural values and assumptions about what makes a good story, a good person, and a fair, just world. The current study focuses specifically on an analysis of U.S. audience evaluations of sexual assault perpetrator (not victim) stories. This focus is intended to be representative of cases in which people might learn that someone has been accused, but never get to hear the alleged victim’s side of the story. The overarching research question asks: does the strength of system justification differentially predict evaluations of high- and low-status perpetrators (and the victims), depending on perpetrator narrative atonement? Audience evaluations of the perpetrator and victim were assessed along several dimensions, including perceived blame or responsibility for the event, perceived event severity, stigma, likability, positive personality traits, and empathic concern. Hypotheses follow below.

#### Hypothesis 1: Evaluations of sexual assault perpetrator

Participant system justification will predict more favorable evaluations of a *high-status* perpetrator when his narrative contains low (vs. medium/high) atonement (Hypothesis 1a) and of a low-status perpetrator as his atonement increases from low to medium to high (Hypothesis 1b).

#### Hypothesis 2: Evaluations of sexual assault victim and event (high-status perpetrator)

Participant system justification will predict less favorable evaluations of a sexual assault victim and less perceived event severity when the high-status perpetrator tells a low (vs. medium/high) atonement narrative (Hypothesis 2). We do not have a priori hypotheses about how medium and high atonement among high-status perpetrators will relate to victim or event evaluations. Examining the latter will be exploratory.

#### Hypothesis 3 (exploratory): Evaluations of sexual assault victim and event (low-status perpetrator)

We do not have a priori hypotheses about how low-status perpetrator atonement and participant system justification will predict victim or event evaluations.

## Materials and method

Study methodology was pre-registered on Open Science Framework (OSF) prior to data collection (https://osf.io/nzb26). All materials, analysis code, and data are on OSF (https://osf.io/76rn9/).

### Participants

To obtain a broad, diverse group of participants and to gather evidence that findings hold while controlling for sample differences, we recruited three samples of U.S. adults (*N* = 895). One sample was recruited via the Psychology Participant Pool at a northwestern public university (*n* = 301) and two via the online platform Prolific. We requested that Prolific participants be census-matched for age and race and that the gender identity of participants be roughly representative of the US population (*n*_1_ = 296 and *n*_2_ = 298). A detailed summary of participant demographic characteristics by sample can be found in Table 1.

**Table 1:** Demographic characteristics of participants by sample.

Characteristic	Total sample (*N* = 895)	University Sample (*n* = 301)	National sample 1 (*n* = 296)	National sample 2 (*n* = 298)
	*N* (%)	*n* (%)	*n* (%)	*n* (%)
Gender				
Man	378 (42.23)	92 (30.56)	141 (47.64)	145 (48.66)
Woman	482 (53.85)	187 (62.13)	150 (50.68)	145 (48.66)
Nonbinary or Genderqueer	23 (2.57)	15 (4.98)	3 (1.01)	5 (1.68)
Preferred to self-describe	5 (0.56)	4 (1.33)	0 (0.00)	1 (2.01)
Preferred not to answer	4 (0.45)	2 (0.66)	1 (0.34)	1 (0.34)
****	3 (0.34)	1 (0.33)	1 (0.34)	1 (2.01)
Sexual Identity				
Straight or Heterosexual	648 (72.40)	174 (57.81)	246 (83.11)	228 (76.51)
Bisexual	155 (17.32)	82 (27.24)	30 (10.14)	43 (14.43)
Gay	19 (2.12)	6 (1.20)	5 (1.69)	8 (2.68)
Lesbian	22 (2.46)	12 (3.99)	4 (1.35)	6 (2.01)
Queer	22 (2.46)	13 (4.7)	4 (1.35)	5 (1.68)
Preferred not to answer	10 (1.12)	6 (1.20)	1 (0.34)	3 (1.01)
Asexual	6 (0.67)	2 (0.66)	1 (0.34)	3 (1.01)
Preferred to self-describe	10 (1.12)	5 (1.66)	4 (1.35)	1 (2.01)
****	3 (0.34)	1 (0.33)	1 (0.34)	1 (2.01)
Race				
White	670 (74.86)	243 (80.40)	212 (71.62)	215 (72.15)
Black or African American	101 (11.28)	11 (3.65)	42 (14.19)	48 (16.11)
Asian	64 (7.15)	17 (5.65)	21 (7.09)	26 (9.73)
Native Hawaiian or Other Pacific Islander	2 (0.22)	0 (0.00)	0 (0.00)	2 (0.67)
American Indian or Alaska Native	4 (0.45)	2 (0.66)	2 (0.68)	0 (0.00)
2 or more races	41 (4.58)	20 (6.64)	16 (5.41)	5 (1.68)
Latinx	96 (10.72)	49 (16.28)	20 (6.76)	27 (9.06)
****	0 (0.00)	0 (0.00)	0 (0.00)	0 (0.00)
	*M* (range)	*M* (range)	*M* (range)	*M* (range)
Age	30.60 (18–82)	19.90 (18–40)	44.36 (18–82)	27.71 (18–76)

*Note*.

**** denotes participants who provided an answer not represented by the available categories.

Our decisions regarding sample size were made both practically (Prolific requires a minimum sample size of 300 to recruit a nationally representative sample) and statistically (with the G*Power software tool [19]). Given alpha at .05, power at .90, and anticipated small-to-medium effect sizes (*f*^2^) for the regression analyses, *n* = 300 participants per sample is in the mid-range between being powered to detect a small-to-medium effect.

#### Inclusion and exclusion criteria

Inclusion criteria were U.S. adults aged 18 and over with English proficiency to complete the surveys. Additionally, data were included in analyses only if participants passed validity checks (three Likert-type attention check questions embedded throughout the survey). For failing one or more such questions, cases were excluded from the first (*n* = 14) and second (*n* = 9) Prolific samples and the university sample (*n =* 48). We also excluded the data of 4 university cases who did not complete the survey in a single sitting.

### Procedures

Our university’s Human Research Protections Program approved the study protocol (#4427EX21). The recruitment period for this study was from 25 Oct 2021 to 11 Feb 2022. Participants completed this approximately 30-minute online study on Qualtrics.com. This study used an electronic consent process. Participants read the consent form prior to participation and were given the opportunity to ask questions about the research via email or phone, and to save/print a copy of the consent form. Participants indicated their informed consent to participate electronically, prior to proceeding to the first page of the survey. Prolific participants received a financial incentive ($4.50 USD) upon valid completion of the survey. University students received course credit for study participation (alternative credit options were available). Participants were randomly assigned to 1 of 2 status conditions (*n*_low_ = 448, *n*_high_ = 447) and 1 of 3 atonement conditions (*n*_low_ = 291, *n*_med_ = 296, *n*_high_ = 308). They read the corresponding passages and then completed study surveys.

### Perpetrator status manipulation

In both status conditions, participants were presented with the same picture of a young white man in his 20’s, referred to as Cody, then read a 91-word paragraph describing Cody’s life, matched in all details except for the description of various markers of his socioeconomic status (low or high). For example, in the low-status condition, Cody “attended a large public high school in one of [the city’s] more economically depressed neighborhoods.” In the high-status condition, he “attended a private college preparatory high school in one of [the city’s] wealthier neighborhoods.” This manipulation was piloted in a pre-registered pilot study and found to adequately affect perceptions of Cody’s status (see S1 File for the pilot study method and results, and S2 File for the complete status manipulation stimuli).

### Perpetrator narrative atonement manipulation

After the status manipulation, participants read a first-person narrative about Cody’s sexual encounter with a woman named Laura and her accusation that he sexually assaulted her (the baseline narrative). We named the accused perpetrator “Cody” and the victim “Laura” based on an analysis of names with a neutral socioeconomic status connotation and with gendered and racialized connotations of belonging to a white male and white female [20]. Cody then concludes his narrative in one of three possible ways, with narrative structure and syntax kept as similar as possible, except for the content reflecting a low, medium, or high level of atonement for his actions. The baseline narrative and atonement manipulation were piloted in two pre-registered pilot studies (see S1 File). A description of each atonement level is below (to read the narratives in full, see S2 File).

#### Narrative 1: Low atonement

To demonstrate lack of responsibility-taking in this narrative, we included elements of a response common to accused perpetrators: Deny, Attack, Reverse Victim and Offender roles (DARVO) [18]. Cody denies wrongdoing and sees himself as the victim (“She’s blaming me…I was really worried about this screwing up my reputation”). His emotional experience consists of anger, defensiveness, and hostility toward Laura (e.g., “[I] told her it [the accusation] was bullshit”). Consistent with his lack of responsibility-taking, Cody does not apologize in this narrative. Instead, he focuses on meeting his own needs [14]. Following Laura’s accusation, he blocks her number and continues life as normal, toward preserving his reputation (“it’s her problem, not mine”).

#### Narrative 2: Medium atonement

Narrative 2 demonstrates a middling amount of responsibility-taking for the sexual assault. Cody admits feeling responsible, but he never fully admits that he sexually assaulted her (“[…] I guess I did ignore a lot that night”). Despite admitting that “I kept going anyway” on the night of the assault, Cody remains preoccupied with his own emotional distress (shame, depression, and avoidance) [14]. Cody says, “I was so ashamed” and “I feel like I don’t deserve to see anyone.” He self-isolates at home, expresses hopelessness about the possibility of change, and fails to engage in personal growth (“I don’t know how I’m gonna recover from this”). Cody’s apology in Narrative 2 is self-focused, emphasizing the distress and regret he feels over his and Laura’s interaction [15]. Cody texts Laura to tell her “I’m sorry and that it’s killing me inside that she hates me now.”

#### Narrative 3: High atonement

In Narrative 3, Cody takes full responsibility for his behavior, acknowledging that he sexually assaulted Laura. While he repeats some lines from Narrative 2 (“[…] I just kept going”) that reflect admission of wrongdoing, what distinguishes the two narratives is Cody’s direct acknowledgement of, concern over, and empathy with Laura’s suffering in Narrative 3. Here, his primary emotion is guilt, an emotion associated with growth following perpetration [14], and his apology is other-focused: he focuses on Laura’s experience, not his own distress (“I […] told her how sorry I was for hurting her”). He demonstrates remorse, but also recognition that he can move forward in a positive way. His actions (asking a woman if she is okay when she seems uncomfortable on a date) demonstrate a desire to right his wrongdoings and prevent harm to others, i.e., full atonement.

### Participant system justification (SJ)

Given that our manipulation of status reflected differences in the perpetrator’s financial income, resources, and influence, we used the Economic System Justification Scale [21], 17 items measuring belief that economic inequality is justified (e.g., “If people work hard, they almost always get what they want”; 1 = *strongly disagree* to 7 = *strongly agree*). Participant responses were averaged across the 17 items (Cronbach’s α = .87), with higher scores on the composite variable (range 1–7) indicating stronger belief in economic SJ.

### Participant evaluations of perpetrator, victim, and event

A complete list of survey items can be found in S1 Table. As stated in our pre-registration, for each dependent variable (DV), we combined the corresponding scale items into a single, mean-scored composite variable if Cronbach’s α > .60 for the items.

#### Stigma

Stigmatizing attitudes were assessed with five items regarding participant desire to engage in relationships of varying closeness with victim or perpetrator (1 = *not at all* to 7 = *very much*) [22, 23]. Stigma items for perpetrator (α = .96) and victim (α = .95) were reverse-coded such that higher scores indicate *more* stigma.

#### Likeability

Perceived likeability was assessed via agreement with the same five statements such as “I would like to get to know Laura/Cody” (1 = *strongly disagree* to 5 = *strongly agree*) about the victim (α = .66) and perpetrator (α = .85) [12].

#### Positive personality traits

Perceived positive personality traits were assessed with an adapted version of the Ten-Item Personality Inventory (TIPI) [24]. Participants indicated level of agreement with the same 10 items, such as “Cody/Laura is calm and emotionally stable” (1 = *strongly disagree* to 7 = *strongly agree*), about victim (α = .71) and perpetrator (α = .74). In the adapted scale, there are two items, one positively worded and one negatively worded (reverse-scored), corresponding with each of the Big Five traits: extraversion, emotional stability, agreeableness, conscientiousness, and openness.

#### Participant empathy

Empathy was assessed according to endorsement of feeling six empathy-related adjectives (e.g., “compassionate”) toward victim (α = .92) and perpetrator (α = .93) from 1 = *not at all* to 7 = *very much* [22, 25].

#### Event severity

Perceived severity of the sexual assault was assessed per agreement with 5 statements regarding the magnitude of the event such as “This event should be taken seriously” (1 = *strongly disagree* to 7 = *strongly agree*). Two negatively worded items were reverse-scored before all 5 items (α = .91) were combined into a single variable.

#### Perceived responsibility for the event

Perceptions of relative perpetrator vs. victim responsibility for the sexual assault was assessed on a zero-sum slider scale from 0 (*Laura’s responsibility*) to 10 (*Cody’s responsibility*).

#### Supplemental measures

Our pre-registered study included five additional DVs: perpetrator and victim responsibility (each on a unidimensional scale), distress toward perpetrator and victim, and perceived blame for the assault (zero-sum scale). For concision, we describe these DVs and results under S3 File, rather than the main text. The overall pattern of findings remains the same with or without these additional measures.

### Data cleaning and coding

Prior to conducting inferential tests of our hypotheses, data were inspected for normality and outliers. For outlying values >|3| SD from the mean, we recoded the outlier to +/-3SD from the mean, using a winsorization approach to retain datapoints but decrease their extremity [26, 27]. In total, 5 DVs had outlying values, ranging from 2 to 19 outlying values per DV; no cases were multivariate outliers.

### Analysis plan

Analyses were conducted in SPSS and R Studio. The overarching prediction in this study was that participant system justification (SJ) and perpetrator atonement would interact to predict more positive evaluations of perpetrators, but crucially, that the interaction would happen one way for the high-status perpetrator condition (greater SJ, lower atonement) and another way for the low-status perpetrator condition (greater SJ, higher atonement). To test the proposed interactive effect on each DV, our pre-registered approach was to conduct regression analyses separately for the high-status perpetrator condition, and the low-status perpetrator condition. For each hierarchical regression model (run separately for high-status and low-status perpetrator conditions), Step 1 consisted of two reverse Helmert-contrast-coded variables accounting for the effect of the three samples: one compared the two national samples, and another compared the national samples vs. university sample. In Step 2, main effects of participant SJ and perpetrator atonement were added to the model and *R*^2^ assessed (*R*^2^_Step2_). Two Helmert-contrast-coded variables accounted for the effect of the three levels of atonement: one compared medium vs. high atonement, and another low vs. the average of medium and high atonement. In Step 3, the hypothesized SJ X atonement interactions were added to model and *R*^2^ assessed (*R*^2^_Step3_). Cohen’s *f*^2^ is the effect size measure for linear models such as the hierarchical multiple regression performed here, with suggested guidelines of 0.02, 0.15, and 0.35 for small, medium, and large effect sizes (*f*^2^), respectively [28]. The formula for Cohen’s *f*^2^ for the overall regression model is $f^{2}=\frac{R_{Step3}^{2}}{1-R_{Step3}^{2}}$ and for the hypothesized SJ x atonement interaction effects only is $f^{2}=\frac{R_{Step3}^{2}-R_{Step2}^{2}}{1-R_{Step3}^{2}}$ [28].

Although we employed a 3-way experimental design and it might seem intuitive to have specified a single overall 3-way statistical model, we did not pre-register that we would do so, for several reasons. First, it is difficult to describe and visualize 3-way interaction effects, especially when one of the three independent variables is continuous (vs. categorical), and we did not want to present readers with highly abstracted statistical results. Second, testing separate statistical models for the high-status and low-status perpetrator conditions was most consistent with our conceptualization of status. When developing our research questions, we separately considered the case of a perpetrator who is high in status, and of a perpetrator who is low in status. We did not propose to compare high- versus low-status perpetrator scenarios against each other, as would be the approach in a 3-way statistical model that enters perpetrator status as a 2-level main effect in the model (and as a component of 2- and 3-way interaction effects).

Lastly, to enable insight into the replicability of our findings, we collected three samples of data. In our pre-registration, we proposed to assess overall support for our hypotheses with a “meta-analysis” of the tests of our hypotheses in each of the three samples, as done in a recent vignette-based experimental study by McLean and colleagues [12]. However, after collecting the data (but before analyzing it), we learned of another approach to handling multiple samples of data: a fixed-effects “mega-analysis” approach [29, 30]. This is the approach described in the first paragraph of this section, where the effects of sample are entered as predictors in the first step of each hierarchical regression model. Mega-analysis uses the full raw pool of data (in this case, data from the three samples) and statistically controls for between-sample heterogeneity in the first step of the regression models, allowing insight into the replicability of results and robustness of effect sizes [31]. As such, we departed from our pre-registration in using a mega-analytic (vs. meta-analytic) approach to the three samples of data. Per the American Psychological Association, “deviations from the original [preregistration] plan are likely to occur; what is important is disclosing them in the final report,” as we do here [32]. We chose to deviate from the preregistered approach because the mega-analytic approach accomplishes an equivalent aim to the meta-analytic approach, but is much less statistically complex, providing more digestible statistical results for readers.

## Results

Table 2 provides descriptive statistics and Table 3, intercorrelations among variables. Condensed hierarchical regression model statistics are presented in Table 4 (perpetrator evaluations), Table 5 (victim evaluations), and Table 6 (event evaluations). For the complete hierarchical regression model statistics, please refer to S4 File. Table 7 presents an at-a-glance summary of results. Sample (national vs. university) was sometimes a statistically significant predictor in the regression models. Given that some sample differences are expected due to random error, we are reluctant to interpret such differences, especially in the absence of a priori hypotheses. A summary of trends in sample differences is provided at the end of Results. All statistical tests of our hypotheses control for the effect of sample, if any.

**Table 2:** Descriptive statistics for perpetrator and victim evaluations by atonement and status.

	Perpetrator Atonement					
	Low Atonement		Medium Atonement		High Atonement	
Perpetrator Status	*M*	*SD*	*M*	*SD*	*M*	*SD*
	*Evaluations of Perpetrator*					
Low-Status Perpetrator						
Stigma	4.75	1.68	4.49	1.55	4.68	1.42
Likeability	2.55	0.87	2.60	0.84	2.89	0.82
Positive Personality Traits	3.00	0.58	3.12	0.48	3.37	0.47
Empathy for Perpetrator	2.32	1.21	2.97	1.51	2.95	1.44
High-Status Perpetrator						
Stigma	5.12	1.52	4.80	1.53	4.87	1.52
Likeability	2.45	0.92	2.54	0.77	2.84	0.81
Positive Personality Traits	2.97	0.59	3.16	0.47	3.34	0.50
Empathy for Perpetrator	2.13	1.29	2.65	1.34	2.58	1.38
	*Evaluations of Victim*					
Low-Status Perpetrator						
Stigma	4.16	1.29	3.91	1.32	3.71	1.26
Likeability	2.85	0.73	2.92	0.76	3.12	0.75
Positive Personality Traits	4.01	0.57	4.02	0.55	4.08	0.60
Empathy for Victim	3.66	1.55	4.03	1.63	4.37	1.46
High-Status Perpetrator						
Stigma	4.34	1.30	3.76	1.25	3.63	1.34
Likeability	2.86	0.71	3.04	0.80	3.18	0.71
Positive Personality Traits	4.00	0.52	4.07	0.64	4.18	0.58
Empathy for Victim	3.76	1.63	4.08	1.51	4.47	1.40
	*Evaluations of Event*					
Low-Status Perpetrator						
Event Severity	5.09	1.32	5.40	1.25	5.72	1.01
Perpetrator Rel. Responsibility	5.79	2.56	6.64	2.69	7.06	2.72
High-Status Perpetrator						
Event Severity	5.15	1.33	5.61	1.26	5.91	0.99
Perpetrator Rel. Responsibility	5.93	2.63	6.84	2.58	7.38	2.50

*Note*. Total possible ranges for each scale are as follows: Stigma 1–7; Likeability 1–5; Positive Personality Traits 1–5 for perpetrator and 1–7 for victim; Empathy 1–7; Event Severity 1–7; Perpetrator Relative Responsibility 0–10.

**Table 3:** Intercorrelations among study variables.

Variable	1	2	3	4	5	6	7	8	9	10	11
1. System justification	--										
2. Stigma (Perp)	-0.36**	—									
3. Likeability (Perp)	0.306**	-0.786**	—								
4. Positive Personality Traits (Perp)	0.186**	-0.566**	0.726**	—							
5. Empathy (Perp)	0.236**	-0.676**	0.686**	0.556**	—						
6. Stigma (Victim)	0.176**	-0.136**	0.206**	0.146**	0.166**	—					
7. Likeability (Victim)	-0.186**	0.186**	-0.176**	-0.136**	-0.176**	-0.766**	—				
8. Positive Personality Traits (Victim)	-0.256**	0.316**	-0.276**	-0.136**	-0.286**	-0.496**	0.546**	—			
9. Empathy (Victim)	-0.336**	0.366**	-0.346**	-0.206**	-0.136**	-0.636**	0.596**	0.486**	—		
10. Event Severity	-0.386**	0.556**	-0.506**	-0.326**	-0.416**	-0.486**	0.476**	0.476**	0.606**	—	
11. Responsibility (Perpetrator vs. Victim) for Event	-0.256**	0.456**	-0.386**	-0.266**	-0.386**	-0.376**	0.396**	0.386**	0.446**	0.556**	—

**p < .01

**Table 4:** Hierarchical regression models predicting evaluations of high- and low-status perpetrators.

*Perpetrator Evaluation *	High-Status Perpetrator					Low-Status Perpetrator				
Predictors	*b *	*SE *	*p *	*R*^*2*^ *(ΔR*^*2*^*)*	*ΔR*^*2*^ *Sig*.	*b *	*SE *	*p *	*R*^*2*^ *(ΔR*^*2*^*)*	*ΔR*^*2*^ *Sig*.
*Stigma*										
Step 3 Predictors				.17 (.00)	.453				.19 (.01)	.062
Sample RHC1	0.09	0.08	.289			-0.08	0.08	.331		
Sample RHC2	-0.17	0.05	< .001			-0.23	0.05	< .001		
SJ	-0.61	0.08	< .001			-0.59	0.07	< .001		
Atonement HC 1	-0.01	0.08	.918			0.12	0.08	.135		
Atonement HC 2	-0.11	0.05	.016			-0.05	0.05	.268		
SJ x Atonement HC 1	-0.09	0.09	.319			0.10	0.09	.284		
SJ x Atonement HC 2	0.04	0.06	.449			0.11	0.05	.041		
*Likeability*										
Step 3 Predictors				.17 (.01)	.048				.17 (.02)	.025
Sample RHC1	-0.10	0.05	.040			-0.01	0.05	.751		
Sample RHC2	0.10	0.03	< .001			0.08	0.03	.002		
SJ	0.26	0.04	< .001			0.30	0.04	< .001		
Atonement HC 1	0.17	0.04	< .001			0.13	0.05	.004		
Atonement HC 2	0.09	0.03	< .001			0.06	0.03	.019		
SJ x Atonement HC 1	0.04	0.05	.471			-0.05	0.05	.275		
SJ x Atonement HC 2	-0.07	0.03	.019			-0.07	0.03	.015		
*Positive Personality Traits*										
Step 3				.20 (.01)	.030				.17 (.03)	.001
Sample RHC1	-0.08	0.03	.006			-0.03	0.03	.225		
Sample RHC2	0.09	0.02	< .001			0.06	0.02	< .001		
SJ	0.11	0.03	< .001			0.10	0.03	< .001		
Atonement HC 1	0.10	0.03	< .001			0.12	0.03	< .001		
Atonement HC 2	0.10	0.02	< .001			0.08	0.02	< .001		
SJ x Atonement HC 1	0.00	0.03	.875			-0.02	0.03	.526		
SJ x Atonement HC 2	-0.05	0.02	.008			-0.07	0.02	< .001		
*Empathy*										
Step 3				.10 (.00)	.117				.14 (.01)	.760
Sample RHC1	-0.02	0.08	.755			0.02	0.08	.808		
Sample RHC2	0.12	0.04	.007			0.18	0.04	< .001		
SJ	0.34	0.07	< .001			0.35	0.07	< .001		
Atonement HC 1	-0.02	0.07	.785			-0.03	0.08	.729		
Atonement HC 2	0.17	0.04	< .001			0.21	0.04	< .001		
SJ x Atonement HC 1	0.00	0.08	.980			0.01	0.08	.911		
SJ x Atonement HC 2	-0.11	0.05	.038			-0.04	0.05	.461		

*Note*. For each model, Step 1 consisted of the sample variables, Step 2 added the main effects, and Step 3 added the interactive effects. For table concision, only Step 3, the full model, is presented here. Please refer to S4 File for the complete hierarchical regression tables with model Steps 1–3. Sample Reverse Helmert Code 1 (RHC1) is coded nationally representative sample 1 = 1, nationally representative sample 2 = -1, and university sample = 0. Sample Reverse Helmert Code 2 (RHC2) is coded nationally representative sample 1 = 1, nationally representative sample 2 = 1, and university sample = -2. SJ refers to Economic System Justification, which was mean-centered. Atonement HC 1 refers to the Helmert Contrast for atonement conditions which compares medium and high levels of atonement. Atonement HC 2 refers to the Helmert Contrast for atonement conditions which compares the low to the average of the medium and high conditions of atonement.

**Table 5:** Hierarchical regression models predicting evaluations of victims of high- and low-status perpetrators.

*Victim Evaluation*	High-Status Perpetrator					Low-Status Perpetrator				
Predictors	*b*	*SE*	*p*	*R*^*2*^ *(ΔR*^*2*^*)*	*ΔR*^*2*^ *Sig*.	*b*	*SE*	*p*	*R*^*2*^ *(ΔR*^*2*^*)*	*ΔR*^*2*^ *Sig*.
*Stigma*										
Step 3				.11 (.00)	.689				.09 (.00)	.487
Sample RHC1	-0.06	0.07	.455			0.00	0.07	.952		
Sample RHC2	0.18	0.04	< .001			0.16	0.04	< .001		
SJ	0.18	0.07	.012			0.25	0.07	< .001		
Atonement HC 1	-0.05	0.07	.537			-0.12	0.07	.104		
Atonement HC 2	-0.21	0.04	< .001			-0.12	0.04	.006		
SJ X Atonement HC 1	0.02	0.08	.817			-0.06	0.08	.436		
SJ X Atonement HC 2	-0.04	0.05	.408			0.04	0.05	.344		
*Likeability*										
Step 3				.08 (.00)	.944				.08 (.00)	.540
Sample RHC1	0.07	0.04	.103			0.08	0.04	.074		
Sample RHC2	-0.07	0.02	.006			-0.07	0.02	< .005		
SJ	-0.14	0.04	< .001			-0.14	0.04	.001		
Atonement HC 1	0.06	0.04	.179			0.11	0.04	.010		
Atonement HC 2	0.08	0.02	.002			0.06	0.02	.020		
SJ X Atonement HC 1	-0.01	0.05	.770			0.02	0.05	.594		
SJ X Atonement HC 2	-0.01	0.03	.861			-0.03	0.03	.319		
*Positive Personality Traits*										
Step 3				.09 (.00)	.671				.10 (.01)	.133
Sample RHC1	-0.07	0.03	.030			-0.03	0.03	.402		
Sample RHC2	-0.03	0.02	.131			-0.06	0.02	.003		
SJ	-0.16	0.03	< .001			-0.15	0.03	< .001		
Atonement HC 1	0.05	0.03	.155			0.04	0.03	.196		
Atonement HC 2	0.04	0.02	.051			0.01	0.02	.469		
SJ X Atonement HC 1	-0.02	0.04	.530			-0.01	0.03	.693		
SJ X Atonement HC 2	0.01	0.02	.531			-0.04	0.02	.051		
*Empathy*										
Step 3				.14 (.00)	.792				.18 (.01)	.438
Sample RHC1	0.03	0.08	.690			0.09	0.08	.271		
Sample RHC2	-0.13	0.05	.009			-0.17	0.05	< .001		
SJ	-0.50	0.08	< .001			-0.56	0.08	< .001		
Atonement HC 1	0.16	0.08	.056			0.20	0.08	.015		
Atonement HC 2	0.15	0.05	.002			0.18	0.05	< .001		
SJ X Atonement HC 1	-0.06	0.09	.512			0.11	0.09	.246		
SJ X Atonement HC 2	-0.01	0.06	.842			-0.03	0.05	.544		

*Note*. For each model, Step 1 consisted of the sample variables, Step 2 added the main effects, and Step 3 added the interactive effects. For table concision, only Step 3, the full model, is presented here. Please refer to S4 File for the complete hierarchical regression tables with model Steps 1–3. Sample Reverse Helmert Code 1 (RHC1) is coded nationally representative sample 1 = 1, nationally representative sample 2 = -1, and university sample = 0. Sample Reverse Helmert Code 2 (RHC2) is coded nationally representative sample 1 = 1, nationally representative sample 2 = 1, and university sample = -2. SJ refers to Economic System Justification, which was mean-centered. Atonement HC 1 refers to the Helmert Contrast for atonement conditions which compares medium and high levels of atonement. Atonement HC 2 refers to the Helmert Contrast for atonement conditions which compares the low to the average of the medium and high conditions of atonement.

**Table 6:** Hierarchical regression models predicting evaluations of events with high- and low-status perpetrators.

*Event Evaluation*	High-Status Perpetrator					Low-Status Perpetrator				
Predictors	*b*	*SE*	*p*	*R*^*2*^ *(ΔR*^*2*^*)*	*ΔR*^*2*^ *Sig*.	*b*	*SE*	*p*	*R*^*2*^ *(ΔR*^*2*^*)*	*ΔR*^*2*^ *Sig*.
*Severity*										
Step 3				.23 (.00)	.675				.27(.01)	.105
Sample RHC1	0.13	0.06	.041			0.10	0.06	.110		
Sample RHC2	-0.16	0.04	< .001			-0.20	0.04	< .001		
SJ	-0.46	0.06	< .001			-0.52	0.06	< .001		
Atonement HC 1	0.11	0.06	.077			0.18	0.06	.003		
Atonement HC 2	0.19	0.04	< .001			0.16	0.04	< .001		
SJ X Atonement HC 1	-0.06	0.07	.386			0.11	0.07	.093		
SJ X Atonement HC 2	-0.01	0.04	.847			0.05	0.04	.222		
Step 3				.13 (.00)	.811				.12 (.01)	.537
Sample RHC1	0.12	0.14	.406			-0.05	0.15	.758		
Sample RHC2	-0.30	0.08	< .001			-0.23	0.09	.008		
SJ	-0.63	0.14	< .001			-0.71	0.14	< .001		
Atonement HC 1	0.23	0.14	.106			0.24	0.15	.106		
Atonement HC 2	0.38	0.08	< .001			0.36	0.09	< .001		
SJ x Atonement HC 1	0.09	0.16	.577			0.09	0.16	.585		
SJ x Atonement HC 2	0.03	0.10	.738			0.09	0.10	.344		

*Note*. For each model, Step 1 consisted of the sample variables, Step 2 added the main effects, and Step 3 added the interactive effects. For table concision, only Step 3, the full model, is presented here. Please refer to S4 File for the complete hierarchical regression tables with model Steps 1–3. Sample Reverse Helmert Code 1 (RHC1) is coded nationally representative sample 1 = 1, nationally representative sample 2 = -1, and university sample = 0. Sample Reverse Helmert Code 2 (RHC2) is coded nationally representative sample 1 = 1, nationally representative sample 2 = 1, and university sample = -2. SJ refers to Economic System Justification, which was mean-centered. Atonement HC 1 refers to the Helmert Contrast for atonement conditions which compares medium and high levels of atonement. Atonement HC 2 refers to the Helmert Contrast for atonement conditions which compares the low to the average of the medium and high conditions of atonement.

**Table 7:** Summary of results for evaluations of sexual assault perpetrator, victim, and event.

Predictor Main Effects	Perpetrator Evaluations	Victim Evaluations	Sexual Assault Event Evaluated as…
System Justification			
*As participant system justification* ***increases****…*	Less Stigmatized	More Stigmatized	Less Severe
	More Likeable	Less Likeable	Less the Perpetrator’s Responsibility Relative to the Victim’s
	More Positive Personality Traits	Less Positive Personality Traits	
	More Empathic Concern	Less Empathic Concern	
Perpetrator Atonement			
*As atonement* ***increases****…*	Less Stigmatized (High-Status Perpetrators only)	Less Stigmatized	More Severe
	More Likeable	More Likeable	More the Perpetrator’s Responsibility Relative to the Victim’s
	More Positive Personality Traits	—	
	More Empathic Concern	More Empathic Concern	

*Note*. All findings consistent across high- and low-status perpetrator conditions unless otherwise noted.

### High-status perpetrator condition: Evaluations of the perpetrator (Hypothesis 1a)

Hypothesis 1a was that participant SJ will interact with perpetrator atonement to predict more favorable evaluations of a high-status perpetrator (less stigma, more likeability, more positive personality traits, more empathy) when his narrative contains low (vs. medium/high) atonement.

#### Stigma toward high-status perpetrator

Hypothesis 1a was not supported. Instead, there were main effects. Higher participant SJ predicted less stigma toward the high-status perpetrator. And as his atonement increased from low to medium/high, stigma lessened. Overall model effect size was medium (*f*^2^ = 0.20).

#### Likeability of high-status perpetrator

**Fig 1:**
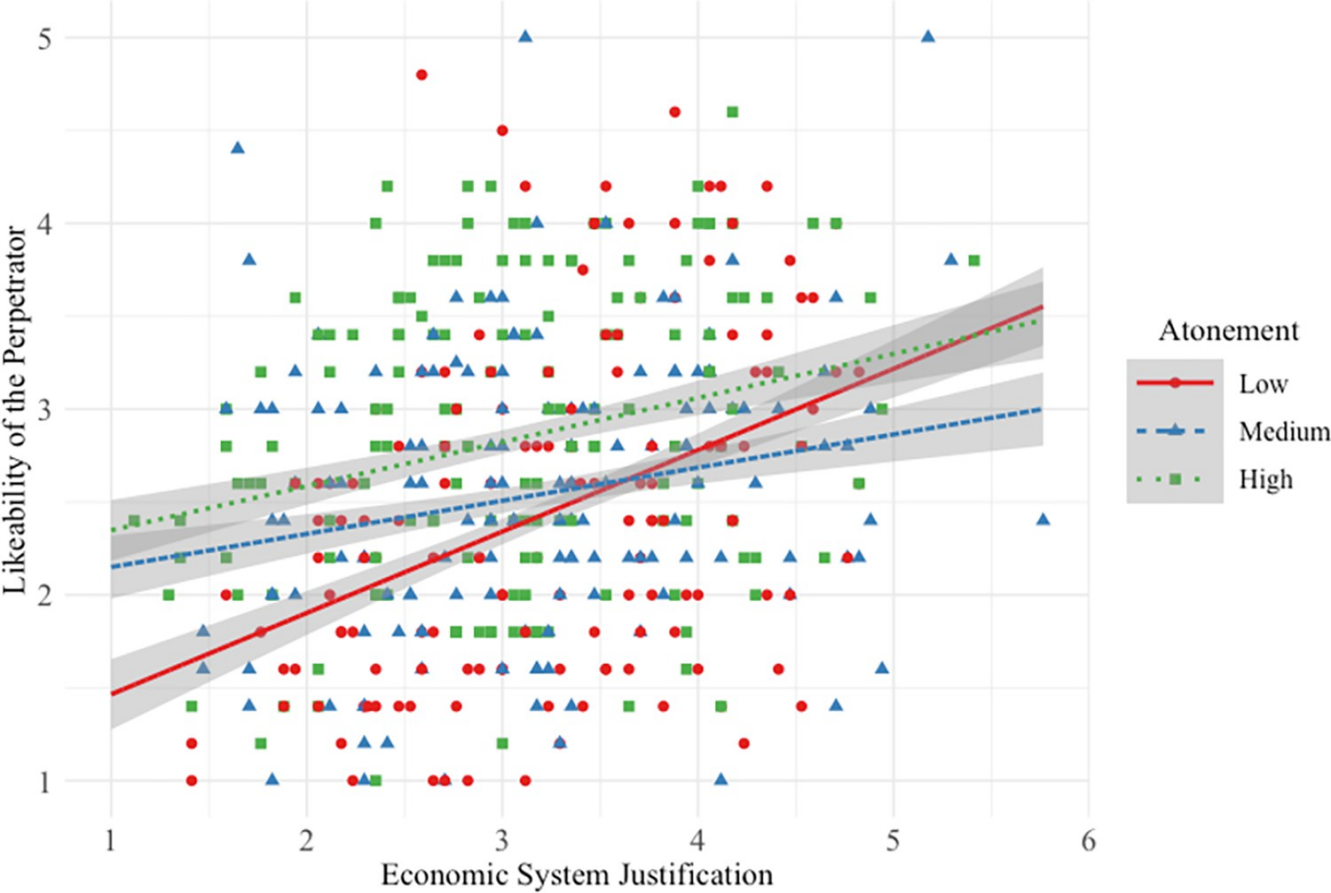
Main and interactive effects of system justification and atonement on high-status perpetrator likeability.

Hypothesis 1a was not supported, in that the interactive effect between SJ and low atonement was not driven by participants higher in SJ, but rather by participants lower in SJ (see Fig 1).

Participants *lower* in SJ reported that the high-status perpetrator was more likeable when his atonement was medium/high versus low. This interactive effect was small (*f*^2^ = 0.01) compared to the strong main effect of SJ on likeability: overall, the higher a participant’s SJ, the more likeable they perceived perpetrators to be. And as atonement increased from low to medium/high and from medium to high, participants rated the high-status perpetrator as more likeable. Overall model effect size was medium (*f*^2^ = 0.20).

### Positive personality traits of high-status perpetrator

**Fig 2:**
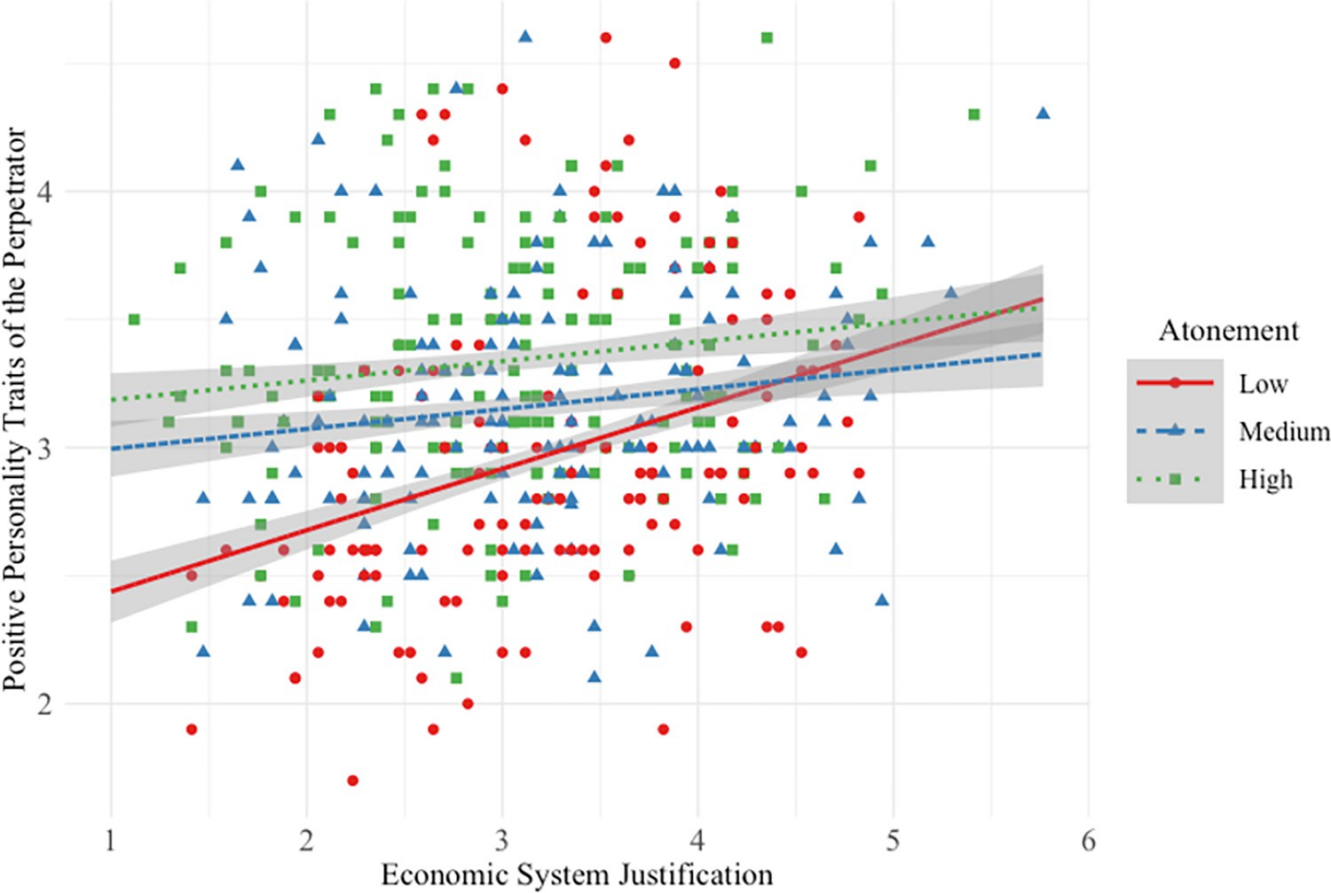
Main and interactive effects of system justification and atonement on high-status perpetrator positive personality traits.

Hypothesis 1a was not supported. Participants *lower* in SJ rated the perpetrator’s personality more positively when his atonement was medium/high (vs. low; see Fig 2).

This interactive effect was small (*f*^2^ = 0.01) compared to the strong main effect of participant SJ on positive personality traits. And as atonement increased from low to medium/high and from medium to high, participants perceived the high-status perpetrator to have more positive personality traits. Overall model effect size was medium-to-large (*f*^2^ = 0.25).

### Empathy for high-status perpetrator

**Fig 3:**
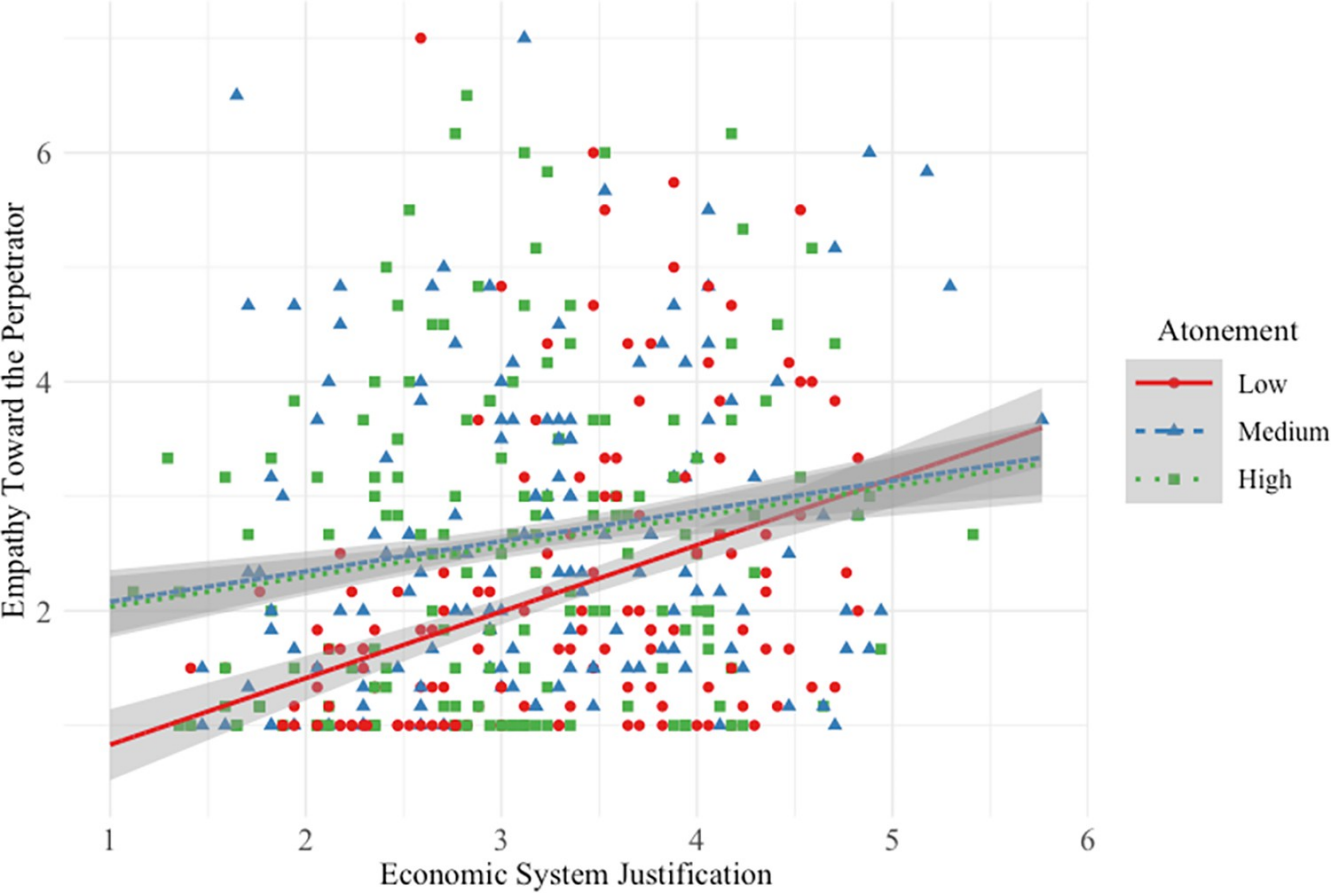
Main and interactive effects of system justification and atonement on empathy for the high-status perpetrator.

Hypothesis 1a was not supported. Participants *lower* in SJ endorsed more empathy for the high-status perpetrator when his atonement was medium/high (vs. low; see Fig 3).

This interactive effect was very small (*f*^2^ = 0.001) compared to the strong main effect of SJ on empathy. As with previous measures, there was a main effect of perpetrator atonement. As it increased from low to medium/high, participants reported more empathy. Overall model effect size was small-to-medium (*f*^2^ = 0.11).

### High-status perpetrator condition: Evaluations of the victim (Hypothesis 2)

Hypothesis 2 was that participant SJ would interact with atonement to predict less favorable evaluations of the victim when the high-status perpetrator tells a low (vs. medium/high) atonement narrative. The hypothesis was not supported for stigma, likeability, positive personality traits, nor empathy. Instead, there was a main effect of SJ: the higher a participant’s SJ, the more they stigmatized the victim; the less likeable they perceived her to be; the lower her positive personality traits; and the less their empathy for her. There was also a main effect of high-status perpetrator atonement: as it increased from low to medium/high, participant stigma toward the victim decreased; she was perceived as more likeable; and empathy for her increased. Overall model effect sizes for victim evaluations were small-to-medium (*f*^2^_stigma_ = 0.12; *f*^2^_like_ = 0.09; *f*^2^_personality_ = 0.10; *f*^2^_empathy_ = 0.16).

### High-status perpetrator condition: Evaluations of the event (Hypothesis 2)

Hypothesis 2 was that SJ would interact with atonement to predict less serious evaluations of the event when the high-status perpetrator tells a low (vs. medium/high) atonement narrative. The hypothesis was not supported for perceived severity of the event nor perpetrator (vs. victim) responsibility for the assault. Instead, there were main effects of SJ: the higher a participant’s SJ, the less severe they perceived the assault by the high-status perpetrator and the less responsibility they assigned to him (vs. to victim). There was also a main effect of high-status perpetrator atonement: as it increased from low to medium/high, participants perceived the assault as more severe and assigned more responsibility to perpetrator (vs. victim). Overall model effect size for event severity was large (*f*^2^ = 0.30) and for perpetrator relative responsibility was medium (*f*^2^ = 0.15).

### Low-status perpetrator condition: Evaluations of the perpetrator (Hypothesis 1b)

Hypothesis 1b was that higher participant SJ will interact with perpetrator atonement to predict more favorable evaluations of a low-status perpetrator (less stigma, more likeability, more positive personality traits, more empathy) as his narrative atonement increases.

#### Stigma toward low-status perpetrator

**Fig 4:**
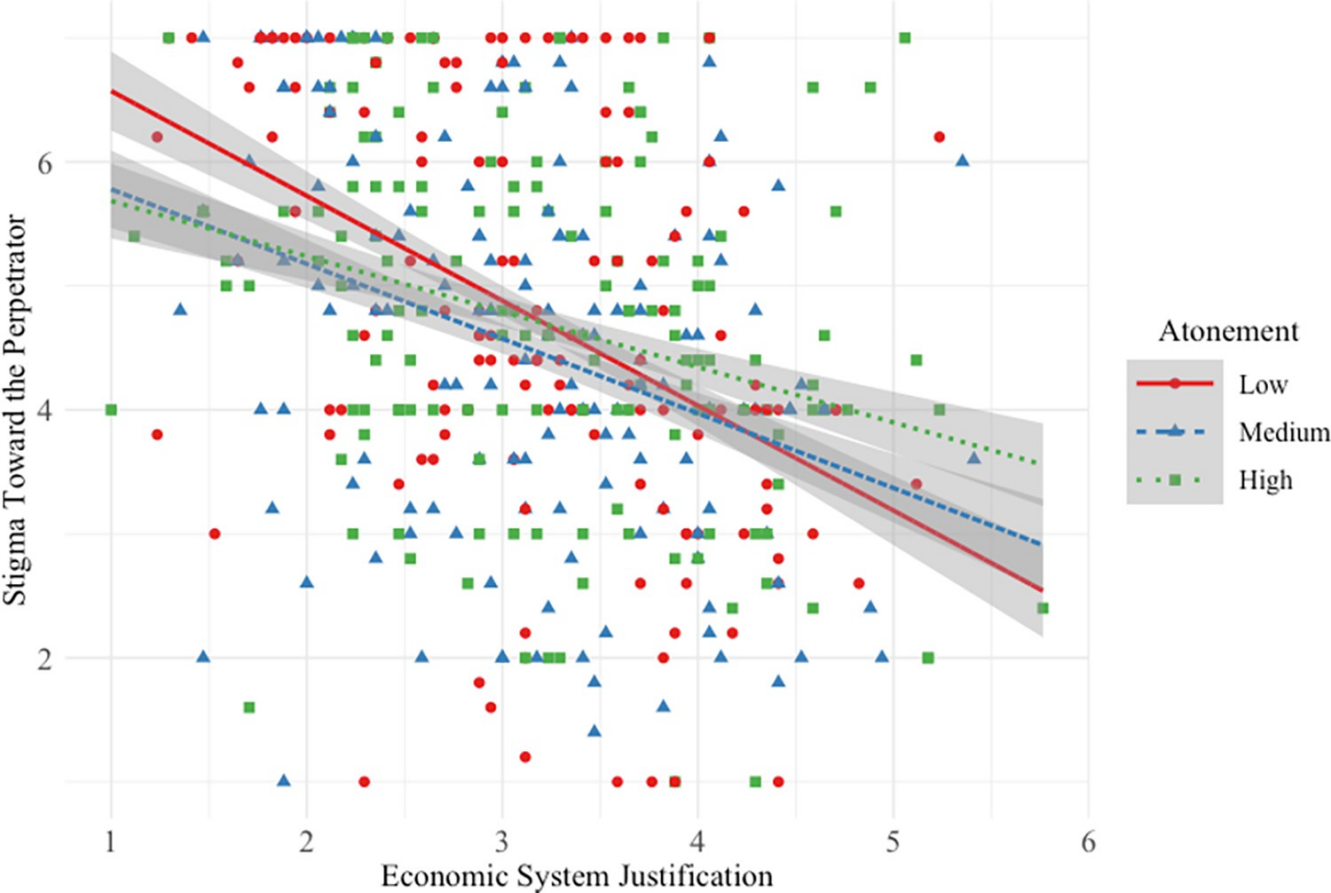
Main and interactive effects of system justification and atonement on stigma toward the low-status perpetrator.

Hypothesis 1b was not supported. Participants *lower* in SJ reported less stigma toward the low-status perpetrator when his atonement was medium/high versus low (see Fig 4).

This interactive effect was small (*f*^2^ = 0.01) when compared to the strong main effect of SJ on stigma: overall, the higher a participant’s SJ, the less they stigmatized low-status perpetrators. There was no main effect of atonement. Overall model effect size was medium-to-large (*f*^2^ = 0.23).

#### Likeability of low-status perpetrator

**Fig 5:**
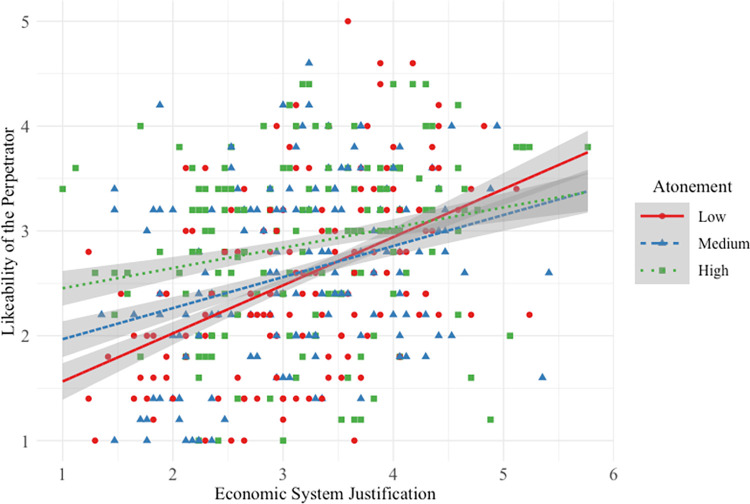
Main and interactive effects of system justification and atonement on low-status perpetrator likeability.

Hypothesis 1b was not supported. Participants *lower* in SJ reported that the low-status perpetrator was more likeable when his atonement was medium/high (vs. low; see Fig 5).

This interactive effect was small (*f*^2^ = 0.02) compared to the strong main effect of SJ: overall, the higher a participant’s SJ, the more likeable they rated perpetrators. And as low-status perpetrator atonement increased from low to medium/high and from medium to high, participants rated him more likeable. Overall model effect size was medium-to-large (*f*^2^ = 0.20).

#### Positive personality traits of low-status perpetrator

**Fig 6:**
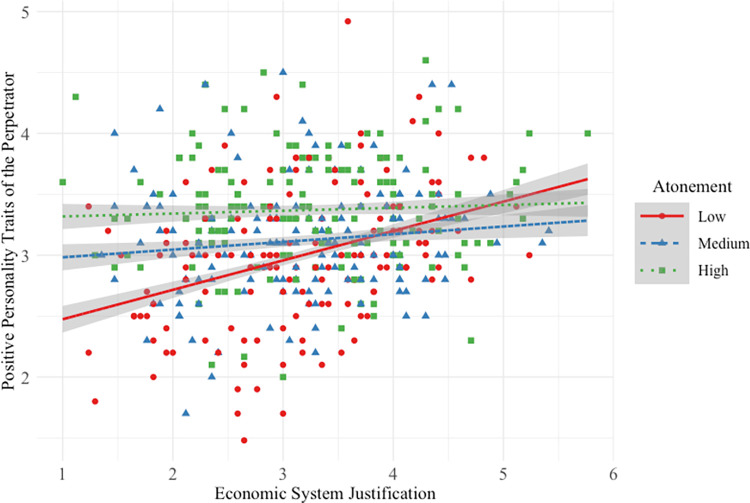
Main and interactive effects of system justification and atonement on low-status perpetrator positive personality traits.

Hypothesis 1b was not supported. Participants *lower* in SJ perceived the perpetrator’s personality more positively when his atonement was medium/high (vs. low; see Fig 6).

This interactive effect was small (*f*^2^ = 0.04) compared to the main effects of SJ and atonement on perceptions of low-status perpetrator positive personality traits. As perpetrator atonement increased from low to medium/high and from medium to high, participants perceived him to have more positive personality traits. Overall model effect size was medium-to-large (*f*^2^ = 0.20).

#### Empathy for low-status perpetrator

Hypothesis 1b was not supported. Instead, there were main effects of SJ and atonement. The higher a participant’s SJ, the greater their empathy for the low-status perpetrator. But as low-status perpetrator atonement increased from low to medium/high, empathy for him increased. Overall model effect size was medium (*f*^2^ = 0.16).

### Low-status perpetrator condition: Evaluations of the victim (Hypothesis 3, exploratory)

There was a main effect of SJ: higher SJ predicted more victim stigma; less likeability; less positive personality traits; and less empathy. There was a main effect of low-status perpetrator atonement. As atonement increased from low to medium/high, participants stigmatized the victim less; liked her more; and reported greater empathy. As atonement increased from medium to high, participants liked the victim more and reported more empathy. Overall model effect sizes for victim evaluations were medium (*f*^2^_stigma_ = 0.10; *f*^2^_like_ = 0.09; *f*^2^_personality_ = 0.11; *f*^2^_empathy_ = 0.22).

### Low-status perpetrator condition: Evaluations of event (Hypothesis 3, exploratory)

There were main effects of SJ: higher SJ predicted *less* perceived event severity and *less* perpetrator (vs. victim) responsibility. There were also main effects of low-status perpetrator narrative atonement. As atonement increased from low to medium/high and from medium to high, the assault was perceived as more severe. And as atonement increased from low to medium/high, participants ascribed more responsibility to perpetrator (vs. victim). Overall model effect size for event severity was large (*f*^2^ = 0.37) and for perpetrator relative responsibility was medium (*f*^2^ = 0.14).

### Summary of sample effects

The two national samples tended not to differ to a statistically significant degree on most evaluations of perpetrator, victim, and event. However, the national samples together almost always differed from the university sample. University (vs. national) participants generally perceived the perpetrator more negatively and victim more positively. University participants stigmatized the perpetrator more and liked and empathized with him less. Mirroring this, they stigmatized the victim less, and liked and empathized with her more. University participants also perceived the event as more severe and assigned more relative responsibility to the perpetrator.

## Discussion

In this experimental study, the story told by a white male perpetrator of sexual assault–specifically, his degree of atonement for his actions–had a direct impact on audience evaluations of him, the female victim, and the event. Greater perpetrator atonement boosted favorable evaluations of perpetrator *and* victim, regardless of perpetrator status. Second, greater participant system justification consistently predicted more favorable evaluations of the perpetrator, less favorable evaluations of the victim, and lower perceived severity of the assault. Third, the influence of participant system justification on evaluations of perpetrator, victim, and event was for the most part fixed, consistent across levels of perpetrator status and atonement. The only exception was that greater perpetrator atonement softened some, but not all, of low-system-justifying individuals’ relatively negative evaluations of the perpetrator. Below, we unpack the ways that these overarching findings do and do not fit with our hypotheses, which were driven by an integration of social-personality, narrative, and trauma psychology theories.

### The role of perpetrator status and participant system justification

That the effects of participant system justification and perpetrator atonement on evaluations were similar across high- and low-status perpetrator conditions was a departure from our hypotheses. Per system justification theory, we had anticipated that participants high in system-justifying tendencies would evaluate high-status perpetrators more favorably when they proudly deny wrongdoing, and low-status perpetrators more favorably when they humbly atone for their actions. One possible explanation for the minimal role of perpetrator status is that when participants evaluated the vignette protagonist for his response to an assault accusation, his socioeconomic status may have been overshadowed by other salient and privileged aspects of his identity (i.e., his gender and race). Participants high in system justifying tendencies may have favored the perpetrator more across the board because he is white and heteronormatively male, and therefore already high in status (particularly in relation to his female victim) regardless of his socioeconomic status. Consequently, our socioeconomically oriented operational definition of a “low status” perpetrator may have nonetheless reflected a relatively “high status” position based on mainstream cultural distinctions of power and privilege.

Furthermore, in the present study, the few interactive effects between system justification and atonement were driven by differences in atonement condition exclusively among participants *low* rather than high in system justification. Low system justifiers are less motivated to preserve the status quo and therefore tend to be lower in rape myth acceptance [33] as well as various sexist ideologies (e.g., hostile, benevolent, modern forms of sexism) [34]. Our patterns suggest that low-system justifying participants softened their negative evaluations of *both* the low- and the high-status perpetrator when he engaged in some degree of atonement over his transgressions. In the case of the low-status perpetrator condition, perhaps low-system justifiers recognized that his lowly economic position in society was part of a larger inequitable system and attempted to provide some form of psychological compensation by rewarding his atonement with more favorable evaluations. In the high-status perpetrator condition, perhaps low-system justifiers appreciated the perpetrator’s atonement as a sort of restitution for the social power he holds. It is worth noting, however, that the tempering effect of atonement was mild: overall perceptions of perpetrators were still more negative among low- (vs. high-) system-justifying participants. Nonetheless, atonement seems to “help” perpetrators on some measures if the audience is low in system-justifying tendencies.

More generally, the consistent main effects of participant system justification on evaluations of the perpetrator, victim, and incident support the power and breadth of individual differences in motivation to maintain the status quo. This is especially noteworthy given that our operationalization of system justification was specific to economic hierarchies (logical, given the success of our status manipulation in our pilot test). Our findings suggest that the beliefs and assumptions of those high in *economic* system justification tendencies extend to other contexts in which inequality and exploitation are legitimized in U.S. culture: specifically, the context of gender-based violence. That is, psychological investment in maintaining an unequal *economic* status quo may also reflect justification of a patriarchal status quo that frames sexual violence as a misunderstanding for which men are vulnerable to false accusations by women. This is consistent with previous work demonstrating that economic system justification is positively associated with the more general hierarchy-enhancing tenets of opposition to equality and group-based dominance associated with Social Dominance Orientation [21].

### Perpetrator narrative atonement impacts perceptions of perpetrators, victims, and event

Degree of perpetrator atonement had a direct impact on almost every evaluation of the sexual assault and its protagonists. For instance, while the sample was neutral in its perceptions of victim likeability, perceptions dropped below neutral (toward negative) in the low-atonement condition. Audiences also expressed less empathic concern and more stigmatizing attitudes toward victims of low-atonement perpetrators. Low-atonement perpetrators were also perceived as less responsible for the assault. Low atonement was characterized by the white male perpetrator’s hostility in response to the accusation, denial of responsibility, self-focus, and victim-blame. Our findings complement vignette-based research by Harsey and Freyd on how male perpetrator denial and victim-blame tactics damage the credibility of female victims and boost perpetrator credibility [18], and also extend prior research in several ways.

This study extends the latter’s findings from the context of physical assault to sexual assault. While our findings support their conclusions on how perpetrator denial influences perceived credibility, we found that denial does negatively impact some evaluations of the perpetrator. In our study, lower atonement made perpetrators seem *less* likable and *less* possessing of positive personality traits. To some extent, the latter findings are unsurprising, in that the low-atonement narrator is objectively harsh in his hostility and victim-blame. What is remarkable is that even the harsh denials of the low-atonement perpetrator led audiences to find him less responsible for the assault and the victim more responsible and more stigmatized. Our findings suggest that, for audiences to judge white men as more credible narrators than women, these men do not need to be sophisticated in their denials, nor particularly likable: they simply need to deny what happened and cast blame on the female victim instead.

Another pattern worth underscoring for its novel contribution is, inversely, the impact of *greater* perpetrator atonement on more *positive* evaluations of the female victim. Narrative atonement in this study was characterized by admission of wrongdoing, apology, and at the highest level, personal growth. Overall, greater atonement evoked more victim likeability, more empathic concern toward the victim, less stigma, and less perceived victim responsibility and blame for the assault. (Results were similar across low- and high-status conditions and for the most part were independent of participant system justification beliefs). This underscores how much the stories that we tell are not only in our own hands: they are co-authored [35]. The finding that men’s redemptive narratives can boost favorable evaluations of female victims is particularly notable when weighed against recent evidence that sexual assault survivor redemptive narratives are not as well-received by U.S. audiences [17, 36].

### Limitations and future directions

To design a tightly controlled experimental study that manipulates only the variables intended (status and atonement) and supports causal inferences, we invented a fictional protagonist and his narratives, along with a named but otherwise anonymous female victim. In everyday life, though, when people learn of a sexual assault accusation, the accused may be someone who they know and respect, while the victim may be unknown to them, especially when assault comes to light in a public/institutional context where confidentiality rules conceal victim identity. As such, our results may underestimate the degree to which audiences give low-atonement perpetrators the benefit of the doubt. This is one limitation of using an experimental design: it is not possible to present a believable experimental manipulation of atonement by a narrator who is already known and respected by study audiences. To complement our experimental findings, future research can take a case-study approach that surveys audience perceptions of a specific public figure’s narrative in response to accusations of violence.

The male and female vignette protagonists in this study are white and, if heteronormative assumptions are made, heterosexual. As such, replication studies are needed to determine the degree to which our findings would generalize to contexts in which the sexual assault perpetrator and victim are of other races/ethnicities; do not share the same race/ethnicity; or are of varying gender or sexual identities. Furthermore, our findings cannot speak to how *participant* race/ethnicity, economic status, gender, or other dimensions of identity might influence evaluations. Indeed, with so many intersecting dimensions of participant identity to consider, experimental designs may not be ideal for addressing contextual questions of how identity influences evaluations [37]. In an experimental design, each dimension of identity must be reductively categorized as a distinct predictor variable in a quantitative statistical model.

Additionally, the differences in story evaluations across national and university samples in this study suggest that there may be other constructs relevant to understanding evaluations. These include developmental stage, university campus culture, or generational differences. Regarding the latter, the #MeToo movement, along with other societal factors, may have prompted a shift in views on sexuality, gender, and interpersonal violence that is more pronounced among younger generations [38]. Future studies designed with a generational analysis in mind can speak to this issue. Lastly, future research that uses a pre-post design or other longitudinal approach can address questions about whether stigmatizing evaluations of sexual assault victims can be mitigated. Perhaps these evaluations may change naturally over time, or purposefully, with prevention/intervention programs.

## Conclusions

When asked, many survivors of gender-based violence report that they want not for perpetrators to be punished, but rather for perpetrators to acknowledge wrongdoing, apologize, and make amends [39]—in other words, to atone. Per Herman’s theory of survivor-centered justice, when a perpetrator atones publicly (vs. privately), his narrative can prompt the broader community to recognize harm done and locate responsibility with him, rather than with the survivor [39]. When the broader community (e.g., bystanders, community members, representatives of institutions) locates responsibility with the perpetrator, this grants survivors a “moral vindication” that is destigmatizing and potentially healing [39 ^p. 85^, 40]. Our findings provide empirical support for Herman’s argument that atonement prompts the community to locate greater responsibility with the perpetrator (vs. victim), and to stigmatize the survivor less. But our findings also indicate that destigmatizing responses to survivors depend strongly upon audience willingness to question status-quo systemic forces that perpetuate interpersonal violence. To disrupt the status quo, the community must reckon with their own complicity in these forces, to trace its beginning, middle, and end. That story has not yet been written.

## Supporting information

S1 Table Scales, items, and response options for study dependent measures. (PDF)

S1 File Pilot study method and results. (PDF)

S2 File Experimental stimuli. Presents the experimental manipulations of perpetrator status and narrative atonement.
(PDF)

S3 File Pre-registered outcome measures and analyses not included in the main manuscript. (PDF)

S4 File Hierarchical regression tables. Tables summarize complete (i.e., Steps 1–3) hierarchical regression model statistics for evaluations of perpetrator, victim, and event.
(PDF)

## References

[1] Bott S, Guedes A, Ruiz-Celis AP, Mendoza J. Intimate partner violence in the Americas: A systematic review and reanalysis of national prevalence estimates. Rev Panam Salud Publica. 2019; 43. doi: 10.26633/RPSP.2019.26 31093250 PMC6425989

[2] Delker BC. Interpersonal violence in context: A call to consider cultural stigma in theory and research on the psychology of trauma. In: McLean KC, editor. Cultural methods in psychology. Oxford, United Kingdom: Oxford University Press; 2022. pp 356–386.

[3] Knight JL, Giuliano TA, Sanchez-Ross MG. Famous or infamous? The influence of celebrity status and race on perceptions of responsibility for rape. Basic and Applied Social Psychology. 2001 Sep 1;23(3):183–90.

[4] Pica E, Sheahan C, Pozzulo J. “But he’s a star football player!”: How social status influences mock jurors’ perceptions in a sexual assault case. Journal of interpersonal violence. 2020 Oct;35(19–20):3963–85. doi: 10.1177/0886260517713715 29294785

[5] Syed M, McLean KC. Master narrative methodology: A primer for conducting structural-psychological research. Cultural Diversity and Ethnic Minority Psychology. 2023 Jan;29(1):53. doi: 10.1037/cdp0000470 34351177

[6] Ali S, Ohls C, Parker G, Walker R. Rationalizing poverty in New York: Tales from the middle class. Journal of Poverty. 2018 Jul 4;22(4):310–33.

[7] Coulter RW, Mair C, Miller E, Blosnich JR, Matthews DD, McCauley HL. Prevalence of past-year sexual assault victimization among undergraduate students: Exploring differences by and intersections of gender identity, sexual identity, and race/ethnicity. Prevention science. 2017 Aug;18:726–36. doi: 10.1007/s11121-017-0762-8 28210919 PMC5511765

[8] Smith SG, Chen J, Basile KC, Gilbert LK, Merrick MT, Patel N. et al. The National Intimate Partner and Sexual Violence Survey. 2017. Centers for Disease Control.

[9] Jost JT, Banaji MR. The role of stereotyping in system‐justification and the production of false consciousness. British journal of social psychology. 1994 Mar;33(1):1–27.

[10] Napier JL, Mandisodza AN, Andersen SM, Jost JT. System justification in responding to the poor and displaced in the aftermath of Hurricane Katrina. Analyses of social issues and public policy. 2006 Dec;6(1):57–73.

[11] Kay AC, Jost JT, Young S. Victim derogation and victim enhancement as alternate routes to system justification. Psychological Science. 2005 Mar;16(3):240–6. doi: 10.1111/j.0956-7976.2005.00810.x 15733206

[12] McLean KC, Delker B, Dunlop WL, Salton R, Syed M. Redemptive stories and those who tell them are preferred in the US. Collabra: Psychology. 2020 6(1). doi: 10.1525/collabra.369

[13] McAdams DP, Manczak E. Personality and the life story. In: Mikulincer M, Shaver PR, editors. APA handbook of personality and social psychology, volume 4: Personality processes and individual differences; 2015. Washington, DC: American Psychological Association Press. pp. 425–446.

[14] Brennan CL, Swartout KM, Cook SL, Parrott DJ. A qualitative analysis of offenders’ emotional responses to perpetrating sexual assault. Sexual Abuse. 2018 Jun;30(4):393–412. doi: 10.1177/1079063216667917 27591752

[15] Nigro G, Ross E, Binns T, Kurtz C. Apologies in the# MeToo moment. Psychology of Popular Media. 2020 Oct ;9(4):403.

[16] Kay AC, Jost JT. Complementary justice: effects of ‘poor but happy’ and ‘poor but honest’ stereotype exemplars on system justification and implicit activation of the justice motive. Journal of personality and social psychology. 2003 Nov;85(5). doi: 10.1037/0022-3514.85.5.823 14599247

[17] Delker BC, Salton R, McLean KC, Syed M. Who has to tell their trauma story and how hard will it be? Influence of cultural stigma and narrative redemption on the storying of sexual violence. Plos one. 2020 Jun 5;15(6):e0234201. doi: 10.1371/journal.pone.0234201 32502207 PMC7274398

[18] Harsey S, Freyd JJ. Deny, attack, and reverse victim and offender (DARVO): what is the influence on perceived perpetrator and victim credibility?. Journal of Aggression, Maltreatment & Trauma. 2020 Sep 13;29(8):897–916. doi: 10.1080/10926771.2020.1774695

[19] Faul F, Erdfelder E, Buchner A, Lang AG. Statistical power analyses using G* Power 3.1: Tests for correlation and regression analyses. Behavior research methods. 2009 Nov;41(4):1149–60. doi: 10.3758/BRM.41.4.1149 19897823

[20] Johfre SS. What age is in a name? Sociological Science 2020; 7: 367–390. doi: 10.15195/v7.a15

[21] Jost JT, Thompson EP. Group-based dominance and opposition to equality as independent predictors of self-esteem, ethnocentrism, and social policy attitudes among African Americans and European Americans. Journal of Experimental Social Psychology. 2000 May 1;36(3):209–32.

[22] Lebowitz MS, Dovidio JF. Implications of emotion regulation strategies for empathic concern, social attitudes, and helping behavior. Emotion. 2015 Apr;15(2):187. doi: 10.1037/a0038820 25706828

[23] Pescosolido BA, Martin JK, Long JS, Medina TR, Phelan JC, Link BG. “A disease like any other”? A decade of change in public reactions to schizophrenia, depression, and alcohol dependence. American journal of psychiatry. 2010 Nov;167(11):1321–30. doi: 10.1176/appi.ajp.2010.09121743 20843872 PMC4429867

[24] Gosling SD, Rentfrow PJ, Swann WB Jr. A very brief measure of the Big-Five personality domains. Journal of Research in personality. 2003 Dec 1;37(6):504–28.

[25] Batson CD, Polycarpou MP, Harmon-Jones E, Imhoff HJ, Mitchener EC, Bednar LL, et al. Empathy and attitudes: Can feeling for a member of a stigmatized group improve feelings toward the group? Journal of personality and social psychology. 1997 Jan;72(1):105. doi: 10.1037//0022-3514.72.1.105 9008376

[26] Goldberg SB, Babins-Wagner R, Imel ZE, Caperton DD, Weitzman LM, Wampold BE. Threat alert: The effect of outliers on the alliance–outcome correlation. Journal of counseling psychology. 2023 Jan;70(1):8.1. doi: 10.1037/cou0000638 36174188 PMC9822845

[27] Hawkins DM. Identification of outliers (Vol. 11). Boca Raton, FL: Chapman & Hall; 1980. doi: 1007/978-94-015-3994-4

[28] Kabacoff RI. R in Action: Data analysis and graphics with R. Shelter Island, NY: Manning Publications Co; 2011.

[29] Costafreda SG. Pooling fMRI data: Meta-analysis, mega-analysis, and multi-center studies. Frontiers in Neuroinformatics. 2009; 3(33): 1–8. doi: 10.3389/neuro.11.033.2009 19826498 PMC2759345

[30] Gonzalez AM, Odic D, Schmader T, Block K, Baron AS. The effect of gender stereotypes on young girls’ intuitive number sense. PloS one. 2021 Oct 28;16(10):e0258886. doi: 10.1371/journal.pone.0258886 34710140 PMC8553059

[31] Curran PJ, Hussong AM. Integrative data analysis: the simultaneous analysis of multiple data sets. Psychological methods. 2009 Jun;14(2):81. doi: 10.1037/a0015914 19485623 PMC2777640

[32] American Psychological Association. Preregistration standards for research in quantitative psychology [Internet]. Washington, D.C.: American Psychological Association; 2021 [cited 2024 Sep 25]. Available from: https://www.apa.org/pubs/journals/resources/preregistration.pdf

[33] Chapleau KM, Oswald DL. Status, threat, and stereotypes: Understanding the function of rape myth acceptance. Social justice research. 2013 Mar;26:18–41.

[34] Monteith MJ, Hildebrand LK. Sexism, perceived discrimination, and system justification in the 2016 US presidential election context. Group Processes & Intergroup Relations. 2020 Feb;23(2):163–78.

[35] McLean KC. The co-authored self: Family stories and the construction of personal identity. Oxford: Oxford University Press; 2016.

[36] Delker BC, Michel PK, Turner K, McLean KC. Perceptions of physical, sexual, and psychological violence stories: A registered report. Collabra: Psychology. 2022; 8(1): 36330. doi: 10.1525/collabra.36330

[37] Syed M. It’s 2 x 2 designs all the way down: Social psychology’s over-reliance on experiments needlessly restricts diversity in the field [Conference presentation]. SPSP 2021 Virtual Conference; 2021, February 5. https://osf.io/gc3mu/

[38] Delker BC, Michel PK, Fogel CA, Patterson AL, Mize G, Huber T, et al. How do young men narrate the redemption story of a sexual assault perpetrator? European Journal of Psychotraumatology; 15(1):2386829. doi: 10.1080/20008066.2024.2386829 39140396 PMC11328808

[39] Herman JL. Truth and repair: How trauma survivors envision justice. New York City: Basic Books; 2023.

[40] Salter M, Hall H. Reducing shame, promoting dignity: A model for the primary prevention of complex post-traumatic stress disorder. Trauma, Violence, & Abuse. 2022 Jul;23(3):906–19. doi: 10.1177/1524838020979667 33345743

