# Supplementary material for: Perceptions of sexual assault perpetrators, victims, and event depend on system justification beliefs and perpetrator atonement
Source: PLoS One. 2024 Dec 31;19(12):e0311983. doi: 10.1371/journal.pone.0311983 (PMC11687665; doi:10.1371/journal.pone.0311983)
Supplement: S1 Table — (PDF) [file pone.0311983.s001.pdf]

S1 Table. Scales, Items, and Response Options for Study Dependent Measures

Scale	Items	Response Options
Stigmatizing Attitudes (Lebowitz & Dovidio, 2015; Pescosolidio et al., 2010)	How much would you like to… *Work closely on a job with the author of this story*<br>*Live next door to each other*<br>*Spend an evening together socializing*<br>*Become relatives by marriage*<br>*Become friends*	1 = Not at All, 7 = Very Much*
Storyteller Likeability (McLean et al., 2020)	*I would like to get to know this person*<br>*I would like to be friends with this person*<br>*The author of this story is mature*<br>*This person is psychologically healthy*<br>*This person is happy*	1 = Strongly Disagree, 5 = Strongly Agree
Storyteller Positive Personality Traits (Gosling, Rentfrow, & Swann, 2003)		
Extraversion	*This person is extraverted and enthusiastic*<br>*This person is reserved and quiet (reversed)*	1 = Strongly Disagree, 7 = Strongly Agree**
Agreeableness	*This person is critical and quarrelsome (reversed)*<br>*This person is sympathetic and warm*	1 = Strongly Disagree, 7 = Strongly Agree
Conscientiousness	*This person is dependable and self-disciplined*<br>*This person is disorganized and careless (reversed)*	1 = Strongly Disagree, 7 = Strongly Agree
Emotional Stability	*This person is anxious and easily upset (reversed)*<br>*This person is calm and emotionally stable*	1 = Strongly Disagree, 7 = Strongly Agree
Openness	*This person is complex and open to new experiences*<br>*This person is conventional and uncreative (reversed)*	1 = Strongly Disagree, 7 = Strongly Agree

Empathy for Storyteller (Batson et al., 1997; Lebowitz & Dovidio, 2015)	Indicate how much each adjective describes your feelings toward this person:	
Empathy	*Sympathetic, Compassionate, Soft-hearted, Warm, Tender, Moved*	1 = Not at All,<br>7 = Very Much
Filler Words	*Angry, Frustrated, Happy, Joyful, Pleased, Resentful*	
Event Severity	*This event should be taken seriously.*<br>*This event is likely to have a long-term impact on the victim's life.*<br>*The event was not a big deal (reverse-scored)*<br>*Laura is over-reacting (reverse-scored)*<br>*Cody sexually assaulted Laura*	1 = Strongly Disagree,<br>7 = Strongly Agree
Responsibility for the Event	*Overall, who has more responsibility for what happened?* (slider scale question)	0 = Laura<br>10 = Cody

Note. Each scale was administered separately with reference to the perpetrator ("Cody") and victim ("Laura"). Mentions of "this person" or "the author" were replaced with the names "Cody" or "Laura" accordingly in the survey that participants completed.
*In the analyses, stigma scales were reverse-scored such that higher scores on stigma connote more stigmatizing attitudes.
**For the evaluation of Cody's positive personality traits, a 1-5 (rather than 1-7) numeric response scale was provided.
