# Supplementary material for: Perceptions of sexual assault perpetrators, victims, and event depend on system justification beliefs and perpetrator atonement
Source: PLoS One. 2024 Dec 31;19(12):e0311983. doi: 10.1371/journal.pone.0311983 (PMC11687665; doi:10.1371/journal.pone.0311983)
Supplement: S1 File — (PDF) [file pone.0311983.s002.pdf]

S1 File. Pilot study method and results.

Pilot Study 1: Manipulating Status of Perpetrator

Method

Participants

A total of 69 participants were recruited from the student subject pool at a public university to participate in this study. Of the participants, 73.9% identified as women, 20.3% identified as men, and 5.8% identified as non-binary or genderqueer. The subject sample consisted of 72.5% White, 10.1% Asian, 10.1% Multiracial, 5.7% Hispanic/Latinx and 1.4% Black participants. Participant ages ranged from 18 to 37 with a mean age of 19.6.

Procedure

Participants were randomly assigned to one of two status conditions. In both conditions, participants were presented with the same picture of a young White man in his mid-20's who is referred to as Cody throughout this study. Then, participants read a paragraph describing Cody's life, in either high or low status circumstances. Please refer to Table S1 for the high- and low-status vignettes. In order to test whether these vignettes accurately portrayed Cody as a high or low status individual, participants were asked to respond to two questions about Cody's socioeconomic status and education. First, participants were presented with a ladder with 10 rungs, and told that each rung represented people's standing in their community. The rung at the very top represented the wealthiest members of the community, and the rung at the bottom represented the poorest members. Participants were asked to choose the rung that they felt accurately represented Cody's standing in his community. Finally, participants were asked to rate

their agreement with the statement *Cody is well-educated* (1 = *Strongly disagree;* 10 = *Strongly agree*).

Results

To test differences in perceptions of Cody's socioeconomic status, we ran a between-subjects *t*-test, $t(36) = -12.45$, 95% CI [-4.33, -3.12], $p < .001$. Participants in the low socioeconomic status condition reported that Cody had lower socioeconomic status ($M = 4.72$, $SD = 0.96$) than participants in the high socioeconomic status condition ($M = 8.45$, $SD = 0.89$). The size of this effect was large ($d = 4.05$).

To test differences in the perceptions of Cody's education, we ran a between-subjects *t*-test, $t(67) = -8.73$, 95% CI [-3.64, -2.28], $p < .001$. Participants in the low socioeconomic status condition perceived that Cody had a lower level of education ($M = 5.83$, $SD = 1.5$) than participants in the high-status condition ($M = 8.55$, $SD = 1.3$). The size of this effect was large ($d = 2.10$).

Discussion

Our analyses indicated that manipulations of both socioeconomic status and level of education were successful. Participants who read the low-status condition narrative reported that Cody was lower in socioeconomic status and education level compared to participants who read the high-status condition.

Pilot Study 2: Manipulating Narrative Atonement

Method

Participants

One hundred and thirty-eight college students ($N = 138$) were recruited for participation from a mid-size, public, Pacific Northwest University (68.8% female, 19.6% male, 4.3% gender

nonbinary, 4.2 % self-identified with different gender identities, and 2.9% did not classify). Regarding gender identification, participants were not asked explicitly about whether they identified as cisgender, and as such we cannot determine rates of cisgender and transgender individuals in this sample. One participant (n = 1) was removed for taking less than 60 seconds to complete the study (M = 215.84, SD = 590.83). Participants were all age 18 and over (M = 20.33, SD = 5.20) and self-reported race/ethnicity in an open-ended question. Due to the open-ended format of the question, participants self-identified their race/ethnicity with a specificity that made it difficult to maintain their anonymity in a predominantly white sample. For the purposes of the pilot results, we are reporting the race/ethnicity of participants as 68.8% White, 12.9% were a combination of 3 different races/ethnicities and biracial individuals, 10.1% Asian, 6.5% Hispanic/Latinx, and 1.4% did not identify.

Materials

Perpetrator Narratives

The baseline narrative and each of the three narrative endings are written from the first-person perspective of a sexual assault perpetrator. They demonstrate his internal narrative process and actions after being accused of sexual assault. All narratives are written in the casual language of a young man in college and vary in length from 113-172 words. Each pilot participant read the same baseline narrative (see more detail below) and was randomly assigned to read one of the three narrative endings (again, details below).

Baseline Narrative. The baseline narrative will be read by all participants. In it, Cody recalls running into Laura on campus, which jogs his memory of a past sexual encounter he had with her. He reports that they met two months ago and went on two dates; in which the sexual encounter occurred on the second date. The day after the sexual contact, Laura text-messages

Cody to accuse him of sexually assaulting her. Cody also describes how a friend of Laura posted about the accusation on social media warning other girls to stay away from him, and his friends saw the post.

The decision for Cody to run into Laura on campus was intentional to control the timing. Two months have passed since the assault, which arguably would provide adequate time for personal growth and perspective on behalf of the perpetrator after the accusation. The public social media post was added to the baseline narrative because it reflects a common reality on college campuses, which provided us with a catalyst for Cody's behavioral and emotional shifts. The emotional tone in this baseline narrative is neutral in order to avoid participants drawing conclusions about Cody’s emotional experience from this narrative. This is important, as emotional variance is part of the study manipulation.

Narrative One: Low Atonement . In order to demonstrate lack of responsibility-taking in this narrative, we included elements of a response common to accused perpetrators: Deny, Attack, Reverse Victim and Offender roles (DARVO; Harsey & Freyd, 2020). Cody sees himself as the victim: “She’s blaming me… I was really worried about this screwing up my reputation.” The emotional experience demonstrated by the perpetrator in Narrative 1 was angry, defensive, and hostile. Cody says, “I was shocked and then pissed because I would never do something like that.” Since anger is associated with higher victim-blaming (Brennan et al., 2018), we also included lines such as “She was clearly into it, and she never said no” and “She had sex that she changed her mind about.” While anger is also associated with derogatory language, we made the choice to leave this type of language out because there was concern that it was unnecessarily inflammatory.

Consistent with the victim-blaming anger, high denial, and no responsibility-taking, Cody does not apologize nor atone in this narrative. His behavior demonstrates an unwillingness to help Laura and he is exclusively focused on meeting his own needs (Brennan et al., 2018). This is demonstrated through his preoccupation with his reputation following the social media post: "For a while, I was really worried about this screwing up my reputation- that girls wouldn't want to date me, or that a future employer would find out." This is short-lived, however, and ultimately, he refuses to let it slow him down; he states, "I've worked so hard to get where I am and I'm not going to let her accusation ruin my future."

Narrative Two: Medium Atonement . Narrative 2 demonstrates a middling amount of responsibility-taking for the sexual assault. Cody admits feeling responsible, but he is preoccupied with his own emotional distress throughout the narrative; he is especially concerned about his possibly tarnished reputation. He says, "…and now everyone who sees her friend's story will think the same thing about me." He still fails to acknowledge what Laura must be feeling, and he never fully admits that he sexually assaulted her. He says, "I have thought about it so much since then, and I see how much I ignored that night. Even though she didn't say no, she didn't seem that into it, and I just kept going." He registers some awareness of his wrongdoing, but the shame he describes throughout the narrative is overwhelming. He consequently lacks the capacity to acknowledge Laura's pain, or act in a way that will support her or any other victim moving forward. Shame and depression are two common and strongly associated emotions following an accusation (Brennan et al., 2018). We demonstrated Cody's shame throughout the narrative with lines such as "I was so ashamed," and "I feel terrible-so angry and ashamed." Additionally, Cody reports feeling depressed, worthless, and angry at himself; for example, he says "I've been in a depression, not going out, not seeing anyone." We

decided not to include the complexity found by Brennan et al. (2018), as oftentimes when perpetrators experience shame, they will blame the situation. We were concerned that adding in this complexity would blur the lines too much between Narratives 1 and 2, as Narrative 1 includes victim-blaming, and we wanted them to be clearly differentiated.

Cody's apology in Narrative 2 is self-focused and lacking in sincerity. Nigro et al. (2019) provided a framework for constructing a self-focused apology. While other-focused apologies include acknowledgement of the victim's suffering, remorse, and reparation, the self-focused apology is characterized by a simple admission of guilt, regret, and desire to improve the self rather than support the victim. While Cody does not directly apologize to Laura in this narrative, he does admit that what he did was wrong. Still, he fails to center Laura's experiences and instead makes statements like, "I have so much regret for what I did." Brennan et al. (2018) found that shame is more strongly associated with avoidance, rather than with growth. We relied on this observation to differentiate between Narratives 2 and 3; in Narrative 2, Cody isolates and feels hopeless in the wake of the assault. His failure to engage in personal growth is cemented in his final statement: "I've worked so hard to get where I am and now I've thrown it away."

Narrative 3: High Atonement . Narrative 3 describes a perpetrator who takes full responsibility for his behavior in it. Cody fully acknowledges that he sexually assaulted Laura. While he repeats some lines from Narrative 2 ("But I have thought about it so much since then, and I see how much I ignored that night… And I just kept going."), what distinguishes the two narratives is that the third fully acknowledges Laura's suffering, and Cody knows he is responsible for causing it. Cody says, "I can't even imagine what she must have been feeling- but I gotta think there's a lot of pain and isolation." He also has a desire to avoid repeating the same

actions: “It was hard to hear, but I’m glad she told me about her experience because now I know how to be better.”

In Narrative 3, Cody’s primary emotion is guilt, since it has been found that guilt is most strongly associated with growth following perpetration (Brennan et al., 2018). For a while, Cody does not speak directly to Laura in the narrative. After some time, he provides an apology that is other-focused; he feels “sadness” for her and demonstrates remorse. It is important that while this perpetrator feels terrible for his behavior, he also knows that he can move forward from it. Therefore, his behavior shows a desire to right his wrong doings and avoid hurting someone else, I.e., atonement.

Narrative Evaluations

After reading Cody’s narrative, pilot participants were presented with five statements pertaining to the narrative and were asked to indicate their level of agreement to each on a four-point Likert scale (1 = disagree strongly, 2 = disagree mildly, 3 = agree mildly, 4 = agree strongly). These statements were designed to assess whether we had successfully manipulated the presence of an apology, the degree of Cody’s personal growth, and the extent to which he took responsibility for his actions. Prior to pilot data collection, we made an a priori decision rule that if the two pre-specified pairs of items (i.e., those related to growth and those related to responsibility) had inter-item associations of $r > .5$, each pre-specified pair would be combined into a composite index.

In accordance with the a priori analysis plan, we created two composite indices. The *growth* index is comprised of the two growth items — “the man has gained insight about his actions and their impact” and “the man has changed his behavior as a result of what happened,” r (136) = .82, $p < .001$. The *responsibility* index is comprised of the two responsibility items —

"the man is taking responsibility for what happened" and "the man acknowledges that he did something wrong," $r(136) = .75$, $p < .001$. These two indices were used for subsequent hypothesis testing. The fifth statement alone measures our success in manipulating the apology construct ("the man is apologizing for what happened").

All participants were then asked to respond to two open-ended questions: "What would make the story sound more realistic?" and "Is there anyone or anything else to blame for what happened?" Only participants who read Narratives 2 or 3 were additionally asked to evaluate the sincerity of Cody's apology.

Results

Exclusion, Indexes, and Variable Groupings

One participant was excluded from final analysis because they took less than a minute to complete the survey, which demonstrated inattentiveness. According to the a priori analysis plan, Narratives 2 and 3 were treated as a single group to be compared against Narrative 1 in the independent *t*-tests for responsibility and apology. This was done because responsibility and apology were expected in Narratives 2 and 3, but not Narrative 1. Additionally, Narratives 1 and 2 were treated as a single group to be compared against Narrative 3 in the independent *t*-test for growth because growth was expected only in Narrative 3.

Responsibility Hypothesis

Consistent with our hypotheses for responsibility, a *t*-test confirmed that perpetrators were perceived to take significantly more responsibility in Narratives 2 and 3 ($M = 3.27$, $SD = .61$) than in Narrative 1 ($M = 1.38$, $SD = .52$), $t(136) = -18.01$, $p < .001$, 95% CI M_D [-2.09, -1.68]. As expected, mean responses to Narrative 1 fell below the mid-point of the scale, and mean responses to Narratives 2 and 3 fell above the mid-point of the scale (2.5).

Apology Hypothesis

Consistent with our hypotheses for apology, a *t*-test confirmed that participants detected that the perpetrator had apologized significantly more in Narratives 2 and 3 ($M = 2.25$, $SD = .91$) than in Narrative 1 ($M = 1.19$, $SD = .45$), $t(136) = -7.50$, $p < .001$, 95% CI M_D [-1.34, -.78]. While mean agreement with apology in Narrative 1 fell below the mid-point of the scale as expected, mean agreement with apology in Narratives 2 and 3 did not fall above the mid-point of the scale (2.5). This indicates that our manipulation of apology was not sufficiently detected in Narratives 2 and 3.

Growth Hypothesis

A final *t*-test confirmed our third hypothesis and indicates that perpetrators were perceived to demonstrate significantly more growth in Narrative 3 ($M = 3.45$, $SD = .56$) than in Narratives 1 and 2 ($M = 2.18$, $SD = 1.05$), $t(136) = -7.61$, $p < .001$, 95% CI M_D [-1.59, -0.93]. Mean perceived growth in Narrative 1 falls below the mid-point of the scale (2.5) and mean perceived growth in Narrative 3 falls above the mid-point of the scale, as expected. However, mean perceived growth in Narrative 2 falls above the mid-point of the scale with a mean of 3.02 ($SD = .67$). A follow up analysis of the difference in perceived growth between Narratives 2 and 3 shows that perceived growth is significantly greater for the perpetrator in Narrative 3 than the perpetrator in Narrative 2, $t(89) = -3.26$, $p = .002$, 95% CI M_D [0.13, -0.68]. This indicates that our manipulation of the perpetrator's personal growth was more convincing in Narrative 3.

Table 2.1

Responsibility and Apology Descriptive Statistics for Narrative 1 versus Narratives 2 and 3

	Atonement			
	Low	Medium and High	*t*	*df*
Responsibility-Taking	1.38 (.52)	3.27 (.61)	-18.01***	136
Apology	1.19 (.45)	2.25 (.91)	-7.50***	136

. *** = $p < .001$. Standard Deviations appear in parentheses below means.

Table 2.2

Growth Descriptive Statistics for Narratives 1 and 2 versus Narrative 3

	Atonement			
	Low and Medium	High	*t*	*df*
Growth	2.18 (1.05)	3.45 (.56)	-7.61***	136

. *** = $p < .001$. Standard Deviations appear in parentheses below means.

Discussion

While quantitative analyses indicated that we successfully manipulated perceived responsibility taking across all three narratives, we failed to effectively manipulate the degree of perpetrator growth present in Narratives 2 and 3. We will need to revise Narrative 2 to exclude any indication that growth was present. Additionally, because the mean perceived apology falls below the mid-point of the scale in both Narratives 2 and 3, we will need to revise both

narratives to clarify that an apology is present (a self-focused apology in Narrative 2 and an other-focused apology in Narrative 3).

In reviewing the quantitative findings, we believe that two factors facilitated the undesirable outcomes mentioned above. The perpetrator in Narrative 2 may have been perceived as having grown simply because of the amount of time he spent ruminating on the assault. In response to this finding, we will aim to incorporate more self-pity, reduce indicators of victim-directed remorse, and control for time spent ruminating about the assault across all three narratives. The presence of an apology most likely went undetected in Narratives 2 and 3 because Cody did not directly apologize to Laura. In response to this finding, we incorporated direct apologies from Cody in all three narratives and remove signs of self-focused apology remorse in the Narrative 3 apology.

Participants responded to an open-ended question asking them what would make the story sound more realistic. Inductive thematic analysis was conducted on participant responses to this question across all conditions. What follows is a discussion of three primary themes and specific coded suggestions that we believe should be incorporated into the narrative revisions. A fourth primary theme indicated that no change was necessary and has been excluded from the following discussion.

More Information

First, many participants reported that more information would make the story more realistic. Specifically, requests were made regarding Laura's side of the story and an omniscient third person telling of the alleged assault. We understand the desire for an objective account of the assault, however, participants indicated that the purpose of receiving more detail was for them to decide whether an assault had indeed occurred. We believe that the ambiguity of the

event itself is both theoretically and practically supported. The “he-said-she-said” framing of sexual assault accusations is ecologically valid and reflected by the lack of detail in these narratives. Additionally, elements such as perpetrator likability, credibility, and authenticity will all be measured as direct outcomes of the way that Cody discusses the assault in the final study. For these reasons, we have chosen not to incorporate either Laura’s side of the story nor an objective telling the assault.

Participants also indicated that hearing Cody’s response to Laura’s text would add realism to the story. We have decided to incorporate Cody’s direct response to Laura in the revised narratives in response to this feedback and to strengthen the apology manipulation.

Cody’s Realism as a Character

Second, concerns about the authenticity of Cody’s character emerged. Participants expressed that his language was too formal for a college-aged man. It was also recommended to include more of Cody’s emotional processing, both to increase the authenticity of his character and to further explicate how Cody became motivated to change or not (depending on condition). In order to address this, we heightened Cody’s emotional response by incorporating more explicit feeling statements and swear words. Similarly, we adapted Cody’s language to include more slang and contractions, and overall attempted to make his tone more colloquial. To demonstrate more of Cody’s internal processing, we increased his elaboration on how his feelings and attitudes toward the accusation and victim changed over time.

Unrealistic Confrontation

Third, participants had several concerns about the way the main plot points of the baseline narrative transpired. Many participants doubted the realism of Laura directly confronting Cody about the assault. Participants suggested, based on similar situations they were

aware of, that either the confrontation would be precipitated by an accidental run-in, or that it would not happen at all. Additionally, participants felt that the timeline for the confrontation was too short, and that it was unrealistic for Laura to text him the morning after the assault. The literature on delayed disclosure (Ullman & Fillipas, 2001; Koss et al., 1991) and the reluctance that sexual assault victims feel to use the labels "assault" or "rape" (Bondurant, 2001; Koss, 1985) supports the decision to lengthen the timeline of events. We revised the baseline narrative so that Cody reaches out to Laura weeks after the sexual assault to ask her for another date. Laura then tentatively suggests that Cody assaulted her, indicating that she too is coming to terms with the traumatic event. We hope that this contact and opportunity for Cody to respond directly to Laura will increase realism and better facilitate an apology in Narratives 2 and 3.

These discussion points have all been incorporated into the revised narratives used for Pilot Experiment 2 (see further detail in the methods section of pilot #2).

Table 2.3

Thematic Analysis Summary

Broad Themes	**Specific Suggestions**	**Incorporated**
More Information	Addition of Laura's perspective	No
	Addition of objective description of sexual encounter	No
	Addition of Cody's response to Laura's accusation	Yes
Cody's Realism as a Character	Change Cody's formal language	Yes
	Describe more of Cody's emotion	Yes
	Describe more about Cody processing the accusation and changing (or not) as a result	Yes
Unrealistic Confrontation	Change the way that Laura confronts Cody about the assault	Yes
	Extend the time between the encounter and the confrontation	Yes
Realistic	n/a	n/a

Pilot Study 3: Manipulating Narrative Atonement

Method

Participants

One hundred and thirty-five college students (*N* = 135) were recruited from a mid-size public university located in the Pacific Northwest (60.7% female, 32.6% male, 2.2% gender non-binary, and 4.4% did not specify). Cis and transgender men and women are included in the male and female frequencies above. Seven participants (*n* = 7) were excluded from the final analysis; six were excluded because they completed the study in under 120 seconds (*M* = 283.46, *SD* = 244.71), and one was excluded because of missing data fields. All participants who specified their age, except one, were over the age of 18 (*M* = 20.01, *SD* = 2.56). Additionally, participants reported their race/ethnicity in an open-ended question (73.3% White/European-American, 8.9% identified as multiracial, 7.4% Asian, 6.7% Hispanic/Latinx, 2.1% were a combination of 3 different races/ethnicities and biracial individuals, and 0.7% did not specify).

Materials

Perpetrator Narratives

All three Narratives in this study are revised versions of those tested in the previous study. For a detailed description of each original narrative and justification of revisions, please refer to the previous discussion section.

Baseline Narrative . The baseline narrative is written from Cody's perspective and it describes, in ambiguous terms, a non-consensual sexual encounter he had with a woman named Laura. Originally, Cody stated "we ended up going back to my place and having sex." In the updated narrative, Cody states "I had sex with her." We made this change to more clearly illustrate that the scenario was non-consensual. Additionally, we made the decision to extend the

time between the assault and the confrontation, which was supported by participant feedback and literature on delayed disclosure (Ullman & Fillipas, 2001). The updated confrontation is initiated by Cody rather than Laura. While in the first version Laura was certain that she was assaulted, the revised narrative portrays her hesitance to call the encounter an assault which is more common among survivors within this timeframe (Koss, 1985).

Narrative One: Low Atonement . In the updated narrative, we strengthened the language that Cody uses to describe his feelings toward the situation. For example, he states, “I was fucking pissed” and “[I] told her it was bullshit.” This aggressive language creates an informal tone, which is more realistic for a college-aged male. Additionally, in the revised narrative, Cody’s lack of responsibility taking is reinforced by his direct response to Laura following her accusation. He blocks her phone number and continues his life as normal. He states, “it’s her problem not mine.”

Narrative Two: Medium Atonement . The revised Narrative 2 includes more emotional language and narrative processing. For example, Cody says, “I’ve just been disgusted with myself” and “[this has] made me question who I am.” As in Narrative 1, this inclusion of more extreme language and detailed cognitive processing makes Cody’s character more realistic. Additionally, we included Cody’s direct response to Laura following her accusation in order to strengthen the apology manipulation. He replies with a self-focused apology emphasizing the distress he feels over their interaction.

Narrative Three: High Atonement . The revised Narrative 3 depicts Cody’s concern for Laura’s wellbeing. He attempts to imagine the impact on Laura instead of ruminating on his own discomfort: “[s]he must feel so violated, and it must be so scary for girls to feel like they can’t say no.” We also decided to include Cody’s direct response to Laura’s accusation. He

replies to her text with an other-focused apology that is attuned to her needs: "I'd be there for her, but I'd understand if she needed space." This response shows the difference between Cody's low level of growth in Narrative 2 and his exceptional growth in Narrative 3.

Narrative Evaluations

Items to assess participant evaluation of the narratives were the same as in Pilot Experiment 1, with the addition of one open-ended response item, "what about the story sounded realistic?" Based on the same a priori decision rule as in Pilot Experiment 1, we computed indices of *growth* (n_{items} = 2; r = .78) and *responsibility* (n_{items} = 2; r = .88) to use in analyses.

Results

Exclusion, Indexes, and Variable Groupings

We started with one-hundred and forty-three participants (N = 143). Six participants were excluded because they took less than 120 seconds to complete the survey, which demonstrated inattentiveness. One participant was removed due to missing data. This left us with a total of N = 135 for analysis.

Responsibility Hypothesis

Consistent with our hypotheses for responsibility taking, a t-test confirmed that there was a significant difference between perceived responsibility taking between participants who read Narrative 1 (M = 1.09, SD = .35) and those who read either Narrative 2 or 3 (M = 3.36, SD = .68), such that those who read Narrative 2 or Narrative 3 perceived greater responsibility taking on the part of the perpetrator (t = 25.85, p < .001). As expected, mean responses to Narrative 1 fell below the mid-point of the scale, and mean responses to Narratives 2 and 3 fell above the mid-point of the scale. As a result of both analyses, we conclude that this variable has been manipulated to the intended degree.

Apology Hypothesis

Consistent with our hypotheses for the presence of an apology, a *t*-test confirmed that there was a significant difference between perceived apology between participants who read Narratives 1 (M = 1.05, SD = .3) and those who read Narratives 2 or 3 (M = 3.2, SD = .86), such that those who read Narrative 2 or Narrative 3 perceived greater apology on the part of the perpetrator than those who read Narrative 1 (t = 21.33, p < .001). Mean responses to Narrative 1 fell below the mid-point of the scale, and mean responses to Narratives 2 and 3 fell above the mid-point of the scale, meaning our changes to the apologies in the narratives were successful, and the apology was detected in Narratives 2 and 3.

Growth Hypothesis

Consistent with our hypotheses for growth, a final *t*-test confirmed that participants who read Narrative 3 perceived significantly more growth (M = 3.43, SD = .68) than participants who read either Narratives 1 or 2 (M = 2.09, SD = 1.01, t = -9.08, p <.001). Mean responses to Narrative 3 fell above the midpoint of the scale, and mean responses to Narratives 1 and 2 fell below the midpoint of the scale. Our changes to the growth level in the narratives were successful in that growth was detected strongly in Narrative 3, but not to a marked degree in Narrative 2. Therefore, we conclude that this variable has been manipulated to the intended degree.

Table 3.1

Atonement Descriptive Statistics for Narrative 1 versus Narratives 2 and 3

	Atonement			
	Low	Medium and High	*t*	*df*
Responsibility-Taking	1.09<br>(.35)	3.36<br>(.68)	25.85***	133
Apology	1.05<br>(.3)	3.2<br>(.86)	21.33***	133

Note. *** = $p < .001$. Standard Deviations appear in parentheses below means.

Table 3.2

Growth Descriptive Statistics for Narratives 1 and 2 versus Narrative 3

	Atonement			
	Low and Medium	High	*t*	*df*
Growth	2.09<br>(1.01)	3.43<br>(.68)	-9.08***	133

Note. *** = $p < .001$. Standard Deviations appear in parentheses below means.

Discussion

Narrative Manipulations

The quantitative analyses of the revised perpetrator atonement narratives indicate that we have successfully manipulated the perceived responsibility taking, degree of perpetrator growth, and perception of apology across all three narratives. The finalized components of atonement at low, medium, and high levels are summarized in Table 3.3 at the end of this document.

Open-Ended Questions

Participants responded to an open-ended question asking what would make the story sound more realistic. Inductive thematic analysis was conducted on participant responses to this question across all conditions. The following is a discussion of the primary themes and specific suggestions from participants. Minor themes and outlier responses were not included in this discussion. Participant feedback will be discussed to further our understanding of how our participant pool generally perceives stories of sexual assault; however, the narratives will not be revised a second time as the variables of perceived responsibility taking, growth, and apology have been manipulated satisfactorily.

Request for More Information

Of the feedback received, a majority of participants stated that more detail in the form of context, further description of the sexual assault, Laura's perspective of the sexual assault, what happened immediately after the sexual assault, and dialogue between Cody and Laura would make the story more realistic. Elements such as perpetrator likability, credibility, and authenticity are all being measured as direct outcomes of the way that Cody discusses the assault. If there was third-person omniscient certainty that an assault had occurred, participants' judgement of Cody would have been skewed. For these reasons, we have chosen not to

incorporate either Laura's side of the story nor an objective telling of the assault. Additionally, we have elected to not further the description of the assault itself, as we recognize the sensitive and potentially triggering content of our narratives. Therefore, we have written the narratives with as much detail as possible, while maintaining ambiguity for participants to form their own conclusions and opinions.

General Feedback

Feedback on Narratives 2 and 3 included statements reflecting the sentiment that Cody's apology, reaction to being accused of sexual assault, and level of responsibility taking are not realistic responses of college age men. As Cody's apology, growth, and responsibility taking are key components of this study, the narratives will not be revised. Responses reflected identification of realism for all three narratives and acknowledgment of the researcher's limitations to provide a longer, more immersive narrative.

Table 3.3

Finalized Experimental Manipulation of Perpetrator Narrative Atonement

	Level of Perpetrator Atonement		
Component of Atonement	Low (Narrative 1)	Medium (Narrative 2)	High (Narrative 3)
Responsibility-taking	None	Admission of wrongdoing with preoccupation on personal distress	Admission of wrongdoing with concern over victim's suffering and desire to improve behavior
Emotion	Anger, denial, hostility	Shame, depression, regret	Guilt, remorse, empathy for victim
Apology type	No apology	Self-focused	Other-focused
Behavior	Focus on personal desires/goals, victim-blaming	Avoidance, social isolation, no growth	Personal growth, attempts to help other victims
