# Supplementary material for: Perceptions of sexual assault perpetrators, victims, and event depend on system justification beliefs and perpetrator atonement
Source: PLoS One. 2024 Dec 31;19(12):e0311983. doi: 10.1371/journal.pone.0311983 (PMC11687665; doi:10.1371/journal.pone.0311983)
Supplement: S2 File — Presents the experimental manipulations of perpetrator status and narrative atonement. (PDF) [file pone.0311983.s003.pdf]

S2 File. Experimental Stimuli

Perpetrator Status

Participants were randomly assigned to read either the high or low status paragraph below, with each being accompanied by the following photo of "Cody":

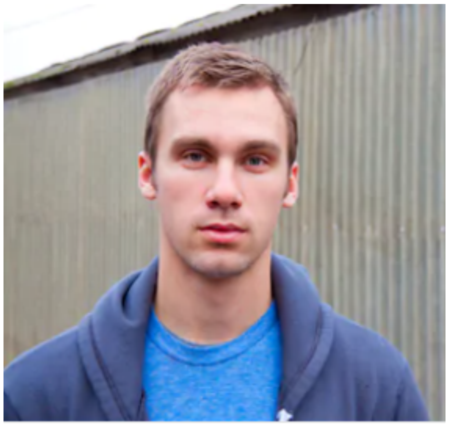

High Status

Cody is a 21-year-old White male from Washington State. He grew up in a relatively big city and attended a private college preparatory high school in one of its wealthier neighborhoods. Attending a well-respected university, he is majoring in pre-law and has spent the past year working a paid internship within his father's prestigious law firm. His luxury SUV makes his travel to class and his internship very comfortable. In his free time, he enjoys playing lacrosse with his friends and skiing with his family at their time share in Switzerland.

Low Status

Cody is a 21-year-old White male from Washington State. He grew up in a relatively big city and attended a large public high school in one of its more economically depressed neighborhoods. Unable to afford in-state tuition at the local public university, he is working full-time alongside his father in a small local construction company. His used pick-up truck makes it easy to haul tools and materials to his job. In his free time, he enjoys playing basketball with his friends and camping with his family at a local state park.

Perpetrator Narrative Atonement

Baseline Narrative

I ran into this girl, Laura, today who I met a couple months ago on Tinder. I thought she was cute and she seemed like she was into me too. On our second date, things were going really well so I kissed her and we ended up going back to my place and I had sex with her. She left before I woke up. A couple weeks later, I hit her up, but she didn't want to go out again. When I asked her why, she said she was really uncomfortable that night and that she was pretty sure it was sexual assault. Then one of my friends told me that he had heard the same thing.

Participants will be randomly assigned to read one of the following three conclusions to "Cody's" story:

Low Atonement

I was fucking pissed because I'd never do something like that. I'm a good guy and that isn't me. I immediately texted her back and told her it was bullshit, and then I blocked her. She was lying. Obviously she was into it, and she never said no. The next day she must have just regretted sleeping with me. She's blaming me because she had sex that she changed her mind about. For a while, I was really worried about this screwing up my reputation- that girls wouldn't want to date me, or that my boss would find out. But honestly, it's not worth my time, since I didn't do anything wrong. It's her problem, not mine. I'm still just doing my thing. I mean, I've worked so hard to get where I am and I'm not gonna let her screw up my future.

Medium Atonement

I was so ashamed. I didn't know what to say, so I avoided it for a couple weeks, and then texted her to tell her that I'm sorry and that it's killing me inside that she hates me now. Ever since then, I've just been disgusted with myself. And now everyone who hears about it will think I'm a monster. I can't stop thinking about it, and I guess I did ignore a lot that night. Even though she didn't say no, she didn't seem that into it, but I kept going anyway. I should've known better. This whole thing has really messed with my head. Made me question who I am. I haven't really been leaving the house. I feel like I don't deserve to see anyone. I always thought of myself as a good guy, but I guess I'm not. I don't know how I'm gonna recover from this.

High Atonement

I felt so guilty that I had hurt her so much. You never wanna be the one that causes that much pain. I texted her right away and told her how sorry I was for hurting her. I said that if she wanted to talk about it more, I'd be there for her, but I'd understand if she needed space. I've thought about it so much over the last couple of weeks, and it's clear how much I ignored her signals that night. In retrospect, she wasn't that into it, and I just kept going. Even though it's been so hard for me, I can't even imagine what she's been feeling. She must feel so violated, and it must be so scary for girls to feel like they can't say no. It was hard to hear, but I'm glad I found out about it, because now I can be better. Last weekend at a bar, I even asked a girl if she was okay, because she seemed a little uncomfortable with her date. I've learned so much about how to be a better person from all this.
