# Supplementary material for: Perceptions of sexual assault perpetrators, victims, and event depend on system justification beliefs and perpetrator atonement
Source: PLoS One. 2024 Dec 31;19(12):e0311983. doi: 10.1371/journal.pone.0311983 (PMC11687665; doi:10.1371/journal.pone.0311983)
Supplement: S3 File — (PDF) [file pone.0311983.s004.pdf]

S3 File. Pre-Registered Outcome Measures and Analyses Not Included in the Main Manuscript.

Overview

For the sake of manuscript concision and audience ability to digest the study findings, we do not report on the following five pre-registered outcome measures and analyses in the main manuscript: perpetrator responsibility (unidimensional scale), victim responsibility (unidimensional scale), participant distress toward the perpetrator, participant distress toward the victim, and perceived perpetrator relative to victim blame for the sexual assault (zero-sum scale). The main effects of participant system justification and perpetrator narrative atonement on these five supplemental measures are consistent with their effects on similar measures reported in the main manuscript, specifically:

- In both the high-status and low-status perpetrator conditions, higher participant system justification scores predicted less perpetrator responsibility (unidimensional scale), more victim responsibility (unidimensional scale), and less blame of the perpetrator relative to the victim (zero-sum scale). There were no effects of participant system justification on participant self-reported distress toward perpetrator or victim.
- In both the high-status and low-status perpetrator conditions, higher perpetrator narrative atonement *increased* perceived perpetrator responsibility (unidimensional scale), *decreased* victim responsibility (unidimensional scale), and *increased* blame of the perpetrator relative to the victim (zero-sum scale). There were no effects of perpetrator narrative atonement on participant self-reported distress toward perpetrator or victim.

The complete description of these measures and the statistical results, tables, and figure can be found below.

Measures

Perceived Responsibility. Perceptions of victim/perpetrator responsibility for the sexual assault was assessed according to agreement with four statements regarding either the victim or the perpetrator's blameworthiness for the event. These items refer to the amount of control each agent had over the situation and if they should be held responsible for the consequences (e.g., "Cody/Laura could have avoided what happened"; 1 = *strongly disagree* to 7 = *strongly agree*). On the same response scale, but only for Cody, participants were asked whether he should be held criminally liable for what happened. Responses to the 4 items (for evaluations of Laura, α = .90) and 5 items (for evaluations of Cody, α = .89) were mean-scored so that each composite variable has the same possible range (1-7).

Participant Distress. Along with participant empathic responses to the victim and perpetrator (reported in the main manuscript), participant distress in response to victim and perpetrator was assessed with six distress-related adjectives (e.g., disturbed, troubled, upset) on a scale from 1 *(not at all*) to 7 (*very much*) (Batson et al., 1997; Lebowitz & Dovidio, 2015). Cronbach's α for the perpetrator distress items was .90, and for the victim, .86.

Blame. Relative blame for the sexual assault event was assessed on a zero-sum slider scale from 0 (*Laura to blame*) to 10 (*Cody to blame*).

Results

Table S13 provides descriptive statistics for participant evaluations of the perpetrator, victim, and event. Table S14 presents intercorrelations among study variables. Tables S17-21 summarize the hierarchical regression model statistics for each dependent measure.

High-Status Perpetrator Condition

Evaluations of the High-Status Perpetrator (Research Question 1, Hypothesis 1a)

Hypothesis 1a was that participant system justification will predict more favorable evaluations of a high-status perpetrator when his narrative contains low (vs. medium/high) atonement. Table S15 provides an overall summary of the results for evaluations of the perpetrator.

Responsibility of the High-Status Perpetrator. Hypothesis 1a was not supported; there was no interactive effect of higher participant system justification and perpetrator low atonement to predict less perpetrator responsibility for the sexual assault. Instead, there were main effects of participant system justification and perpetrator atonement. Higher participant system justification scores predicted less high-status perpetrator responsibility (b = -0.48, SE = 0.06, p < .001). As high-status perpetrator atonement increased from low to medium/high (b = 0.21, SE = 0.04, p < .001) and from medium to high (b = 0.15, SE = 0.06, p = .016), participants perceived him to be more responsible for the assault.

Distress toward the High-Status Perpetrator. Hypothesis 1a was not supported; there was no interactive effect of higher participant system justification and perpetrator low atonement to predict less distress toward the high-status perpetrator. Nor were there any main effects.

Evaluations of the Victim of a High-Status Perpetrator (Research Question 2, Hypothesis 2)

Hypothesis 2 was that participant system justification would predict less favorable evaluations of the victim when the high-status perpetrator tells a low (vs. medium/high) atonement narrative. Table S15 provides an overall summary of the results for evaluations of the victim.

Responsibility of the Victim of a High-Status Perpetrator. Hypothesis 2 was not supported, in that the interactive effect between participant system justification and perpetrator low atonement was not driven by participants higher in system justification, but rather

participants lower in system justification. Participants *lower* in system justification tendencies reported that the victim of the high-status perpetrator was less responsible for the event when perpetrator atonement was high (versus medium), $b = 0.16$, $SE = 0.08$, $p = .038$; see Figure S1). This relative attenuation of victim responsibility was modest when compared to the strong main effect of participant system justification: overall, the higher a participant's system justification tendencies, the more responsible they perceived victims to be ($b = 0.67$, $SE = 0.07$, $p < .001$). There was also a main effect of perpetrator atonement on victim responsibility. As high-status perpetrator atonement increased from low to medium/high ($b = -0.19$, $SE = 0.04$, $p < .001$) and from medium to high ($b = -0.18$, $SE = 0.07$, $p = .010$), participants rated the victim as less responsible for the assault.

Distress toward the Victim of the High-Status Perpetrator. Hypothesis 2 was not supported; there was no interactive effect of higher participant system justification and perpetrator low atonement to predict distress toward the victim. Nor were there any main effects.

Evaluations of the Sexual Assault Event: High-Status Perpetrator Condition (Research Question 2, Hypothesis 2)

Hypothesis 2 was participant system justification would predict less serious evaluations of the event when the high-status perpetrator tells a low (vs. medium/high) atonement narrative. Table S16 provides a summary of results for evaluations of the sexual assault.

Relative Blame for the Event. Hypothesis 2 was not supported; there was no interactive effect of higher participant system justification and perpetrator low atonement. Instead, there were main effects of participant system justification and perpetrator atonement. Higher participant system justification scores predicted less high-status perpetrator blame relative to victim blame ($b = -0.80$, $SE = 0.13$, $p < .001$). As high-status perpetrator atonement increased

from low to medium/high (*b* = 0.39, *SE* = 0.08, *p* < .001), participants blamed the perpetrator more, relative to the victim.

Low-Status Perpetrator Condition

Evaluations of the Low-Status Perpetrator (Research Question 1, Hypothesis 1b)

Hypothesis 1b was that higher participant system justification will predict more favorable evaluations of a low-status perpetrator as his narrative atonement increases.

Responsibility of the Low-Status Perpetrator. Hypothesis 1b was not supported; there was no interactive effect of participant system justification and perpetrator atonement to predict less perpetrator responsibility for the sexual assault. Instead, there were main effects of participant system justification and perpetrator atonement. Higher participant system justification scores predicted less low-status perpetrator responsibility (*b* = -0.42, *SE* = 0.06, *p* < .001). As low-status perpetrator atonement increased from low to medium/high (*b* = 0.18, *SE* = 0.04, *p* < .001) and from medium to high (*b* = 0.15, *SE* = 0.06, *p* = .017), participants perceived him to be more responsible for the assault.

Distress toward the Low-Status Perpetrator. Hypothesis 1b was not supported; there was no interactive effect of higher participant system justification and perpetrator low atonement to predict less distress toward the low-status perpetrator. Nor were there any main effects.

Evaluations of the Victim of a Low-Status Perpetrator (Research Question 3, Exploratory)

In the case of low-status perpetrators, we did not have a priori hypotheses about how participant system justification and perpetrator atonement would predict victim evaluations. As stated in our pre-registration, these are exploratory analyses.

Responsibility of the Victim of a Low-Status Perpetrator. There was no interactive effect of participant system justification and perpetrator atonement to predict victim

responsibility for the sexual assault. There were main effects of both system justification and perpetrator atonement. The higher a participant's system justification tendencies, the more responsible they perceived victims to be (b = 0.66, SE = 0.07, p < .001). There was also a main effect of perpetrator atonement on victim responsibility. As low-status perpetrator atonement increased from low to medium/high (b = -0.13, SE = 0.04, p = .003) and from medium to high (b = -0.29, SE = 0.07, p < .001), participants rated the victim as less responsible for the assault.

Distress toward the Victim of the Low-Status Perpetrator. There were no main effects of participant system justification and perpetrator atonement, nor any interactive effect, on distress toward the victim.

Evaluations of the Sexual Assault Event: Low-Status Perpetrator Condition (Research Question 3, Exploratory)

In the case of low-status perpetrators, we did not have a priori hypotheses about how participant system justification and perpetrator atonement would predict event evaluations. As stated in our pre-registration, these are exploratory analyses.

Relative Blame for the Event. There were main effects of participant system justification and perpetrator atonement, and no interactive effect. Higher participant system justification scores predicted less low-status perpetrator blame relative to victim blame (b = -0.85, SE = 0.12, p < .001). As low-status perpetrator atonement increased from low to medium/high (b = 0.42, SE = 0.08, p < .001) and from medium to high (b = 0.40, SE = 0.13, p = .003), participants blamed the perpetrator more, relative to the victim.

Scales

Table S12

Scales, Items, and Response Options for Additional Study Dependent Measures

Scale	Items	Response Options
Responsibility	*This person is responsible for what happened*<br>*This person is to blame for what happened*<br>*This person could have avoided what happened*<br>*This person had control over what happened*<br>*[Cody only] This person should be held criminally liable for what happened.*	1 = Strongly Disagree<br>7 = Strongly Agree
Relative Blame	*Overall, who is more to blame for what happened?* (slider)	0 = Laura<br>10 = Cody
Distress toward Storyteller (Batson et al., 1997; Lebowitz & Dovidio, 2015)	Indicate how much each adjective describes your feelings toward this person:	
Distress	*Alarmed, Distressed, Disturbed, Troubled, Upset, Worried*	1 = Not at All,<br>7 = Very Much
Filler Words	*Angry, Frustrated, Happy, Joyful, Pleased, Resentful*	

Note. Each scale was administered separately with reference to the perpetrator ("Cody") and victim ("Laura"). Mentions of "this person" or "the author" were replaced with the names "Cody" or "Laura" accordingly in the survey that participants completed.

Table S13

Descriptive Statistics for Additional Perpetrator, Victim, and Event Evaluations by Atonement and Status Conditions

Perpetrator Status	Perpetrator Atonement					
	Low Atonement		Medium Atonement		High Atonement	
	M	*SD*	*M*	*SD*	*M*	*SD*
Low Status						
Perpetrator Responsibility	4.57	1.29	4.97	1.23	5.23	0.98
Victim Responsibility	3.95	1.35	3.84	1.46	3.33	1.51
Distress toward Perpetrator	3.74	1.45	3.88	1.53	3.79	1.56
Distress toward Victim	3.48	1.46	3.51	1.56	3.22	1.43
Perpetrator Relative Blame	5.95	2.46	6.82	2.70	7.54	2.22
High Status						
Perpetrator Responsibility	4.60	1.35	5.09	1.21	5.45	0.99
Victim Responsibility	3.99	1.44	3.59	1.38	3.12	1.38
Distress toward Perpetrator	3.96	1.53	4.07	1.64	4.16	1.56
Distress toward Victim	3.22	1.39	3.48	1.56	3.31	1.40
Perpetrator Relative Blame	6.09	2.58	7.02	2.54	7.65	2.32
Total						
Perpetrator Responsibility	4.59	1.32	5.03	1.22	5.34	0.99
Victim Responsibility	3.97	1.39	3.71	1.43	3.22	1.44
Distress toward Perpetrator	3.85	1.49	3.98	1.58	3.98	1.57
Distress toward Victim	3.35	1.43	3.49	1.56	3.27	1.41
Perpetrator Relative Blame	6.02	2.51	6.92	2.62	7.59	2.27

Note. Total possible ranges for each scale are as follows: Responsibility 1-7; Distress 1-7; Perpetrator Relative Blame 0-10.

Table S14

Intercorrelations among Study Variables

Variable	1	2	3	4	5	6	7	8	9	10	11	12	13	14	15
1. System justification	—														
2. Stigma (Perp)	-0.36**	—													
3. Likeability (Perp)	0.30**	-0.78**	—												
4. Positive Personality Traits (Perp)	0.18**	-0.56**	0.72**	—											
5. Empathy (Perp)	0.23**	-0.67**	0.68**	0.55**	—										
6. Stigma (Victim)	0.17**	-0.13**	0.20**	0.14**	0.16**	—									
7. Likeability (Victim)	-0.18**	0.18**	-0.17**	-0.13**	-0.17**	-0.76**	—								
8. Positive Personality Traits (Victim)	-0.25**	0.31**	-0.27**	-0.13**	-0.28**	-0.49**	0.54**	—							
9. Empathy (Victim)	-0.33**	0.36**	-0.34**	-0.20**	-0.13**	-0.63**	0.59**	0.48**	—						
10. Event Severity	-0.38**	0.55**	-0.50**	-0.32**	-0.41**	-0.48**	0.47**	0.47**	0.60**	—					
11. Responsibility (Perpetrator vs. Victim) for Event	-0.25**	0.45**	-0.38**	-0.26**	-0.38**	-0.37**	0.39**	0.38**	0.44**	0.55**	—				
12. Distress (Perpetrator)	-0.13**	0.24**	-0.27**	-0.22**	-0.15*	-0.18**	0.16**	0.09**	0.29**	0.28**	0.21**	—			

13. Responsibility (Perpetrator) for Event	-0.35**	0.59**	-0.54**	-0.35**	-0.46**	-0.49**	0.48**	0.48**	0.60**	0.77**	0.64**	0.25**	—		
14. Distress (Victim)	0.03	-0.08*	0.05	0.03	0.35**	0.11**	-0.14**	-0.17**	0.13**	-0.03	-0.10**	0.18**	-0.08*	—	
15. Responsibility (Victim) for Event	0.44**	-0.58**	0.49**	0.33**	0.47**	0.47**	-0.48**	-0.46**	-0.53**	-0.66**	-0.62**	-0.24**	-0.68**	0.14**	—
16. Blame (Perpetrator vs. Victim) for Event	-0.31**	0.50**	-0.44**	-0.26**	-0.39**	-0.45**	0.45**	0.43**	0.53**	0.63**	0.65**	0.22**	0.73**	-0.11**	-0.70**

Note. Variables 12-16 are the supplemental variables not included in the main text.
**p* < .05
***p* < .01

Table S15

Summary of Results for Evaluations of Sexual Assault Perpetrator and Victim, with Additional Variables in Bold Font

Predictor Main Effects	Perpetrator Evaluations	Victim Evaluations
System Justification<br><br>*As participant system justification* ***increases****…*	**Less Responsible**	**More Responsible**
	Less Stigmatized	More Stigmatized
	More Likeable	Less Likeable
	More Positive Personality Traits	Less Positive Personality Traits
	More Empathic Concern	Less Empathic Concern
	**Less Distress (Low-Status Perpetrators only)**	**---**
Perpetrator Atonement<br><br>*As atonement* ***increases****…*	**More Responsible**	**Less Responsible**
	Less Stigmatized (High-Status Perpetrators only)	Less Stigmatized
	More Likeable	More Likeable
	More Positive Personality Traits	--
	More Empathic Concern	More Empathic Concern
	**--**	**--**

Note. All findings consistent across high- and low-status perpetrator conditions unless otherwise noted.

Table S16

Summary of Results for Evaluations of Incident, with Additional Variable in Bold Font

Predictor Main Effects	Sexual Assault Incident Evaluated as…
System Justification	
As participant system justification ***increases****…*	Less Severe<br><br>Less the Perpetrator's Responsibility Relative to the Victim's<br><br>**Less Perpetrator Blame Relative to Victim Blame**
Perpetrator Atonement	
As atonement ***increases****…*	More Severe<br><br>More the Perpetrator's Responsibility Relative to the Victim's<br><br>**More Perpetrator Blame Relative to Victim Blame**

Note. All findings consistent across high- and low-status perpetrator conditions.

Hierarchical Regression Tables

Tables refer to evaluations of the perpetrator (S17-S18), victim (S19-S20), and event (S21).

Table S17

Hierarchical Regression Predicting Responsibility of the Perpetrator

Predictor	High-Status Perpetrator					Low-Status Perpetrator				
	b	*SE*	*p*	R^2 (ΔR^2)	ΔR^2 *Sig.*	*b*	*SE*	*p*	R^2 (ΔR^2)	ΔR^2 *Sig.*
Step 1				.04	< .001				.02	.019
Sample RHC1	0.08	0.07	.255			-0.01	0.07	.838		
Sample RHC2	-0.16	0.04	< .001			-0.11	0.04	.005		
Step 2				.23 (.19)	< .001				.16 (.14)	< .001
Sample RHC1	0.04	0.06	.501			0.00	0.06	.991		
Sample RHC2	-0.13	0.04	< .001			-0.08	0.04	.037		
SJ	-0.47	0.06	< .001			-0.42	0.06	< .001		
Atonement HC 1	0.15	0.06	.017			0.15	0.06	.016		
Atonement HC 2	0.21	0.04	< .001			0.18	0.04	< .001		
Step 3				.23 (.00)	.559				.17 (.01)	.691
Sample RHC1	0.04	0.06	.567			0.00	0.06	.971		
Sample RHC2	-0.13	0.04	< .001			-0.08	0.04	.035		
SJ	-0.48	0.06	**< .001**			-0.42	0.06	**< .001**		
Atonement HC 1	0.15	0.06	**.016**			0.15	0.06	**.017**		
Atonement HC 2	0.21	0.04	**< .001**			0.18	0.04	**< .001**		
SJ x Atonement HC 1	0.00	0.07	.977			0.05	0.07	.502		
SJ x Atonement HC 2	0.05	0.04	.281			0.02	0.04	.613		

Note. Sample Reverse Helmert Code 1 (RHC1) is coded nationally representative sample 1 = 1, nationally representative sample 2 = -1, and university sample = 0. Sample Reverse Helmert Code 2 (RHC2) is coded nationally representative sample 1 = 1, nationally representative sample 2 = 1, and university sample = -2. SJ refers to Economic System Justification, which was mean-centered.
Atonement HC 1 refers to the Helmert Contrast for atonement conditions which compares medium and high levels of atonement. Atonement HC 2 refers to the Helmert Contrast for atonement conditions which compares the low to the average of the medium and high conditions of atonement.

Table S18

Hierarchical Regression Predicting Distress toward the Perpetrator

Predictor	High-Status Perpetrator					Low-Status Perpetrator				
	b	*SE*	*p*	R^2 (ΔR^2)	ΔR^2 *Sig.*	*b*	*SE*	*p*	R^2 (ΔR^2)	ΔR^2 *Sig.*
Step 1				.07	< .001				.04	< .001
Sample RHC1	0.16	0.09	.071			0.04	0.09	.660		
Sample RHC2	-0.28	0.05	< .001			-0.20	0.05	< .001		
Step 2				.08 (.01)	.450				.06 (.02)	.009
Sample RHC1	0.15	0.09	.086			0.05	0.09	.593		
Sample RHC2	-0.28	0.05	< .001			-0.18	0.05	< .001		
SJ	-0.09	0.08	.304			-0.26	0.08	.001		
Atonement HC 1	0.03	0.09	.770			-0.03	0.09	.726		
Atonement HC 2	0.06	0.05	.258			0.03	0.05	.531		
Step 3				.08 (.00)	.974				.06 (.00)	.649
Sample RHC1	0.15	0.09	.093			0.05	0.09	.591		
Sample RHC2	-0.28	0.05	< .001			-0.18	0.05	< .001		
SJ	-0.09	0.08	.299			-0.27	0.08	< .001		
Atonement HC 1	0.03	0.09	.760			-0.03	0.09	.704		
Atonement HC 2	0.06	0.05	.261			0.03	0.05	.533		
SJ x Atonement HC 1	0.01	0.10	.910			0.00	0.09	.958		
SJ x Atonement HC 2	0.01	0.06	.843			0.05	0.06	.355		

Note. Sample Reverse Helmert Code 1 (RHC1) is coded nationally representative sample 1 = 1, nationally representative sample 2 = -1, and university sample = 0. Sample Reverse Helmert Code 2 (RHC2) is coded nationally representative sample 1 = 1, nationally representative sample 2 = 1, and university sample = -2. SJ refers to Economic System Justification, which was mean-centered. Atonement HC 1 refers to the Helmert Contrast for atonement conditions which compares medium and high levels of atonement. Atonement HC 2 refers to the Helmert Contrast for atonement conditions which compares the low to the average of the medium and high conditions of atonement.

Table S19

Hierarchical Regression Analyzing Victim Responsibility

Predictor	High Status Perpetrator					Low Status Perpetrator				
	b	*SE*	*p*	R^2 (ΔR^2)	ΔR^2 *Sig.*	*b*	*SE*	*p*	R^2 (ΔR^2)	ΔR^2 *Sig.*
Step 1				.07	< .001				.07	< .001
Sample RHC1	-0.04	0.08	.643			0.07	0.08	.380		
Sample RHC2	0.28	0.05	< .001			0.27	0.05	< .001		
Step 2				.30 (.23)	< .001				.26 (.19)	< .001
Sample RHC1	0.01	0.07	.852			0.05	0.07	.478		
Sample RHC2	0.23	0.04	< .001			0.21	0.04	< .001		
SJ	0.66	0.07	< .001			0.66	0.07	< .001		
Atonement HC 1	-0.19	0.07	.008			-0.29	0.07	< .001		
Atonement HC 2	-0.19	0.04	< .001			-0.12	0.04	.003		
Step 3				.30 (.00)	.109				.27 (.01)	.689
Sample RHC1	0.01	0.07	.915			0.05	0.07	.522		
Sample RHC2	0.23	0.04	< .001			0.21	0.04	< .001		
SJ	0.67	0.07	**< .001**			0.66	0.07	**< .001**		
Atonement HC 1	-0.18	0.07	**.010**			-0.29	0.07	**< .001**		
Atonement HC 2	-0.19	0.04	**< .001**			-0.13	0.04	**.003**		
SJ X Atonement HC 1	0.16	0.08	**.038**			0.07	0.08	.398		
SJ X Atonement HC 2	-0.02	0.05	.733			0.01	0.05	.892		

Note. Sample Reverse Helmert Code 1 (RHC1) is coded nationally representative sample 1 = 1, nationally representative sample 2 = -1, and university sample = 0. Sample Reverse Helmert Code 2 (RHC2) is coded nationally representative sample 1 = 1, nationally representative sample 2 = 1, and university sample = -2. SJ refers to Economic System Justification, which was mean-centered. Atonement HC 1 refers to the Helmert Contrast for atonement conditions which compares medium and high levels of atonement. Atonement HC 2 refers to the Helmert Contrast for atonement conditions which compares the low to the average of the medium and high conditions of atonement.

Table S20

Hierarchical Regression Analyzing Distress Toward the Victim

Predictor	High Status Perpetrator					Low Status Perpetrator				
	b	*SE*	*p*	R^2 (ΔR^2)	ΔR^2 *Sig.*	*b*	*SE*	*p*	R^2 (ΔR^2)	ΔR^2 *Sig.*
Step 1				.02	.001				.00	.495
Sample RHC1	0.25	0.08	.003			0.04	0.09	.617		
Sample RHC2	0.03	0.05	.575			0.05	0.05	.282		
Step 2				.03 (.01)	.266				.01 (.01)	.347
Sample RHC1	0.26	0.08	.002			0.04	0.09	.621		
Sample RHC2	0.02	0.05	.749			0.05	0.05	.279		
SJ	0.10	0.08	.186			-0.01	0.08	.911		
Atonement HC 1	-0.08	0.08	.340			-0.14	0.09	.098		
Atonement HC 2	0.06	0.05	.237			-0.04	0.05	.475		
Step 3				.03 (.00)	.410				.01 (.00)	.842
Sample RHC1	0.26	0.08	.003			0.04	0.09	.657		
Sample RHC2	0.02	0.05	.737			0.05	0.05	.287		
SJ	0.11	0.08	.169			-0.01	0.08	.885		
Atonement HC 1	-0.08	0.08	.365			-0.14	0.09	.097		
Atonement HC 2	0.06	0.05	.213			-0.04	0.05	.465		
SJ X Atonement HC 1	0.11	0.09	.226			0.06	0.09	.559		
SJ X Atonement HC 2	-0.03	0.06	.584			-0.00	0.06	.936		

Note. Sample Reverse Helmert Code 1 (RHC1) is coded nationally representative sample 1 = 1, nationally representative sample 2 = -1, and university sample = 0. Sample Reverse Helmert Code 2 (RHC2) is coded nationally representative sample 1 = 1, nationally representative sample 2 = 1, and university sample = -2. SJ refers to Economic System Justification, which was mean-centered. Atonement HC 1 refers to the Helmert Contrast for atonement conditions which compares medium and high levels of atonement. Atonement HC 2 refers to the Helmert Contrast for atonement conditions which compares the low to the average of the medium and high conditions of atonement.

Table S21

Hierarchical Regression Predicting Perpetrator's Relative Blame for the Incident

Predictor	High-Status Perpetrator					Low-Status Perpetrator				
	b	*SE*	*p*	R^2 (ΔR^2)	ΔR^2 Sig.	*b*	*SE*	*p*	R^2 (ΔR^2)	ΔR^2 Sig.
Step 1				.06	< .001				.03	.002
Sample RHC1	0.33	0.14	.022			0.04	0.15	.800		
Sample RHC2	-0.40	0.08	< .001			-0.30	0.08	< .001		
Step 2				.19 (.13)	< .001				.18 (.15)	< .001
Sample RHC1	0.27	0.13	.047			0.07	0.13	.611		
Sample RHC2	-0.34	0.08	< .001			-0.23	0.08	.003		
SJ	-0.78	0.13	< .001			-0.85	0.12	< .001		
Atonement HC 1	0.25	0.13	.057			0.40	0.13	.003		
Atonement HC 2	0.39	0.08	< .001			0.42	0.08	< .001		
Step 3				.20 (.01)	.290				.18 (.00)	.833
Sample RHC1	0.25	0.14	.066			0.07	0.13	.611		
Sample RHC2	-0.34	0.08	< .001			-0.23	0.08	003		
SJ	-0.80	0.13	**< .001**			-0.85	0.12	**< .001**		
Atonement HC 1	0.26	0.13	.051			0.40	0.13	**.003**		
Atonement HC 2	0.39	0.08	**< .001**			0.42	0.08	**< .001**		
SJ x Atonement HC 1	-0.01	0.15	.948			0.01	0.15	.967		
SJ x Atonement HC 2	0.15	0.09	.116			0.05	0.09	.549		

Note. Relative blame for the sexual assault was assessed on a zero-sum slider scale from 0 (victim to blame) to 10 (perpetrator to blame). Sample Reverse Helmert Code 1 (RHC1) is coded nationally representative sample 1 = 1, nationally representative sample 2 = -1, and university sample = 0. Sample Reverse Helmert Code 2 (RHC2) is coded nationally representative sample 1 = 1, nationally representative sample 2 = 1, and university sample = -2. SJ refers to Economic System Justification, which was mean-centered. Atonement HC 1 refers to the Helmert Contrast for atonement conditions which compares medium and high levels of atonement. Atonement HC 2 refers to the Helmert Contrast for atonement conditions which compares the low to the average of the medium and high conditions of atonement.

Figure S1. Main and Interactive Effects of System Justification and Atonement on Perceived Responsibility of the Victim of a High-Status Perpetrator.

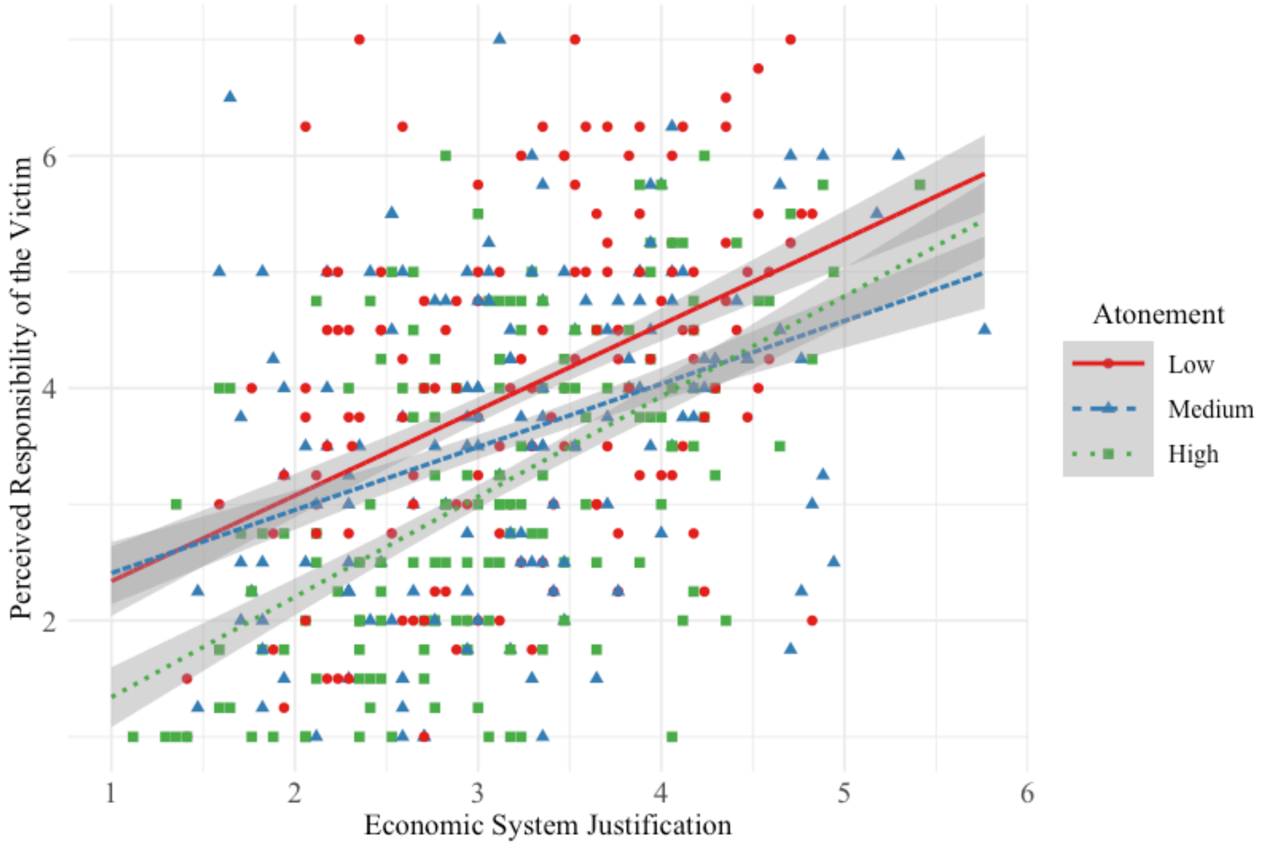

Note. Red circles indicate raw data and red solid line indicates regression line for the low atonement condition. Blue triangles indicate raw data and blue dashed line indicates regression line for the medium atonement condition. Green squares indicate raw data and green dotted line indicates regression line for the high atonement condition.
