# Supplementary material for: Perceptions of sexual assault perpetrators, victims, and event depend on system justification beliefs and perpetrator atonement
Source: PLoS One. 2024 Dec 31;19(12):e0311983. doi: 10.1371/journal.pone.0311983 (PMC11687665; doi:10.1371/journal.pone.0311983)
Supplement: S4 File — Tables summarize complete (i.e., Steps 1–3) hierarchical regression model statistics for evaluations of perpetrator, victim, and event. (PDF) [file pone.0311983.s005.pdf]

S4 File. Hierarchical Regression Tables

Tables summarize regression model statistics for evaluations of the perpetrator (S2-S5), victim (S6-S9), and event (S10-S11).

Table S2

Hierarchical Regression Predicting Stigma Toward the Perpetrator

Predictor	High-Status Perpetrator					Low-Status Perpetrator				
	b	*SE*	*p*	R^2 (ΔR^2)	ΔR^2 *Sig.*	*b*	*SE*	*p*	R^2 (ΔR^2)	ΔR^2 *Sig.*
Step 1				.05	< .001				.06	<.001
Sample RHC1	0.13	0.09	.145			-0.09	0.09	.307		
Sample RHC2	-0.22	0.05	< .001			-0.27	0.05	< .001		
Step 2				.17 (.12)	< .001				.18 (.12)	< .001
Sample RHC1	0.09	0.08	.279			-0.07	0.08	.362		
Sample RHC2	-0.17	0.05	< .001			-0.23	0.05	< .001		
SJ	-0.60	0.08	< .001			-0.57	0.08	< .001		
Atonement HC 1	-0.01	0.08	.943			0.13	0.08	.115		
Atonement HC 2	-0.11	0.05	.019			-0.05	0.05	.285		
Step 3				.17 (.00)	.453				.19 (.01)	.062
Sample RHC1	0.09	0.08	.289			-0.08	0.08	.331		
Sample RHC2	-0.17	0.05	< .001			-0.23	0.05	< .001		
SJ	-0.61	0.08	< .001			-0.59	0.07	< .001		
Atonement HC 1	-0.01	0.08	.918			0.12	0.08	.135		
Atonement HC 2	-0.11	0.05	.016			-0.05	0.05	.268		
SJ x Atonement HC 1	-0.09	0.09	.319			0.10	0.09	.284		
SJ x Atonement HC 2	0.04	0.06	.449			0.11	0.05	.041		

Note. Sample Reverse Helmert Code 1 (RHC1) is coded nationally representative sample 1 = 1, nationally representative sample 2 = -1, and university sample = 0. Sample Reverse Helmert Code 2 (RHC2) is coded nationally representative sample 1 = 1, nationally representative sample 2 = 1, and university sample = -2. SJ refers to Economic System Justification, which was mean-centered. Atonement HC 1 refers to the Helmert Contrast for atonement conditions which compares medium and high levels of atonement. Atonement HC 2 refers to the Helmert Contrast for atonement conditions which compares the low to the average of the medium and high conditions of atonement.

Table S3

Hierarchical Regression Predicting Likeability of the Perpetrator

Predictor	High-Status Perpetrator					Low-Status Perpetrator				
	b	*SE*	*p*	R^2 (ΔR^2)	ΔR^2 *Sig.*	*b*	*SE*	*p*	R^2 (ΔR^2)	ΔR^2 *Sig.*
Step 1				.05	< .001				.03	.001
Sample RHC1	-0.12	0.05	.016			-0.01	0.05	.829		
Sample RHC2	0.12	0.03	< .001			0.10	0.03	< .001		
Step 2				.16 (.11)	< .001				.15 (.12)	< .001
Sample RHC1	-0.10	0.05	.026			-0.02	0.05	.708		
Sample RHC2	0.10	0.03	< .001			0.08	0.03	.002		
SJ	0.25	0.04	< .001			0.29	0.04	< .001		
Atonement HC 1	0.17	0.05	< .001			0.13	0.05	.006		
Atonement HC 2	0.09	0.03	.001			0.06	0.03	.021		
Step 3				.17 (.01)	.048				.17 (.02)	.025
Sample RHC1	-0.10	0.05	.040			-0.01	0.05	.751		
Sample RHC2	0.10	0.03	< .001			0.08	0.03	.002		
SJ	0.26	0.04	< .001			0.30	0.04	< .001		
Atonement HC 1	0.17	0.04	< .001			0.13	0.05	.004		
Atonement HC 2	0.09	0.03	< .001			0.06	0.03	.019		
SJ x Atonement HC 1	0.04	0.05	.471			-0.05	0.05	.275		
SJ x Atonement HC 2	-0.07	0.03	.019			-0.07	0.03	.015		

Note. Sample Reverse Helmert Code 1 (RHC1) is coded nationally representative sample 1 = 1, nationally representative sample 2 = -1, and university sample = 0. Sample Reverse Helmert Code 2 (RHC2) is coded nationally representative sample 1 = 1, nationally representative sample 2 = 1, and university sample = -2. SJ refers to Economic System Justification, which was mean-centered. Atonement HC 1 refers to the Helmert Contrast for atonement conditions which compares medium and high levels of atonement. Atonement HC 2 refers to the Helmert Contrast for atonement conditions which compares the low to the average of the medium and high conditions of atonement.

Table S4
Hierarchical Regression Predicting Positive Personality Traits of the Perpetrator

Predictor	High-Status Perpetrator					Low-Status Perpetrator				
	b	*SE*	*p*	R^2 (ΔR^2)	ΔR^2 *Sig.*	*b*	*SE*	*p*	R^2 (ΔR^2)	ΔR^2 *Sig.*
Step 1				.09	< .001				.03	.001
Sample RHC1	-0.09	0.03	.003			-0.03	0.03	.265		
Sample RHC2	0.10	0.02	< .001			0.06	0.02	< .001		
Step 2				.19 (.10)	< .001				.14 (.11)	< .001
Sample RHC1	-0.09	0.03	.003			-0.03	0.03	.227		
Sample RHC2	0.09	0.02	< .001			0.06	0.02	.001		
SJ	0.10	0.03	< .001			0.09	0.03	< .001		
Atonement HC 1	0.10	0.03	.001			0.12	0.03	< .001		
Atonement HC 2	0.09	0.02	< .001			0.08	0.02	< .001		
Step 3				.20 (.01)	.030				.17 (.03)	.001
Sample RHC1	-0.08	0.03	.006			-0.03	0.03	.225		
Sample RHC2	0.09	0.02	< .001			0.06	0.02	< .001		
SJ	0.11	0.03	< .001			0.10	0.03	< .001		
Atonement HC 1	0.10	0.03	< .001			0.12	0.03	< .001		
Atonement HC 2	0.10	0.02	< .001			0.08	0.02	< .001		
SJ x Atonement HC 1	0.00	0.03	.875			-0.02	0.03	.526		
SJ x Atonement HC 2	-0.05	0.02	.008			-0.07	0.02	< .001		

Note. Sample Reverse Helmert Code 1 (RHC1) is coded nationally representative sample 1 = 1, nationally representative sample 2 = -1, and university sample = 0. Sample Reverse Helmert Code 2 (RHC2) is coded nationally representative sample 1 = 1, nationally representative sample 2 = 1, and university sample = -2. SJ refers to Economic System Justification, which was mean-centered. Atonement HC 1 refers to the Helmert Contrast for atonement conditions which compares medium and high levels of atonement. Atonement HC 2 refers to the Helmert Contrast for atonement conditions which compares the low to the average of the medium and high conditions of atonement.

Table S5

Hierarchical Regression Predicting Empathy Toward the Perpetrator

Predictor	High-Status Perpetrator					Low-Status Perpetrator				
	b	*SE*	*p*	R^2 (ΔR^2)	ΔR^2 *Sig.*	*b*	*SE*	*p*	R^2 (ΔR^2)	ΔR^2 *Sig.*
Step 1				.03	.003				.04	< .001
Sample RHC1	-0.06	0.08	.472			0.03	0.08	.756		
Sample RHC2	0.15	0.04	.001			0.20	0.05	< .001		
Step 2				.10 (.07)	< .001				.13 (.09)	< .001
Sample RHC1	-0.04	0.08	.619			0.02	0.08	.793		
Sample RHC2	0.12	0.04	.008			0.18	0.04	< .001		
SJ	0.33	0.07	< .001			0.34	0.07	< .001		
Atonement HC 1	-0.02	0.07	.840			-0.03	0.08	.714		
Atonement HC 2	0.17	0.04	< .001			0.21	0.04	< .001		
Step 3				.10 (.00)	.117				.14 (.01)	.760
Sample RHC1	-0.02	0.08	.755			0.02	0.08	.808		
Sample RHC2	0.12	0.04	.007			0.18	0.04	< .001		
SJ	0.34	0.07	< .001			0.35	0.07	< .001		
Atonement HC 1	-0.02	0.07	.785			-0.03	0.08	.729		
Atonement HC 2	0.17	0.04	< .001			0.21	0.04	< .001		
SJ x Atonement HC 1	0.00	0.08	.980			0.01	0.08	.911		
SJ x Atonement HC 2	-0.11	0.05	.038			-0.04	0.05	.461		

Note. Sample Reverse Helmert Code 1 (RHC1) is coded nationally representative sample 1 = 1, nationally representative sample 2 = -1, and university sample = 0. Sample Reverse Helmert Code 2 (RHC2) is coded nationally representative sample 1 = 1, nationally representative sample 2 = 1, and university sample = -2. SJ refers to Economic System Justification, which was mean-centered. Atonement HC 1 refers to the Helmert Contrast for atonement conditions which compares medium and high levels of atonement. Atonement HC 2 refers to the Helmert Contrast for atonement conditions which compares the low to the average of the medium and high conditions of atonement.

Table S6

Hierarchical Regression Analyzing Stigma Toward the Victim

Predictor	High Status Perpetrator					Low Status Perpetrator				
	b	*SE*	*p*	R^2 (ΔR^2)	ΔR^2 *Sig.*	*b*	*SE*	*p*	R^2 (ΔR^2)	ΔR^2 *Sig.*
Step 1				.04	< .001				.04	< .001
Sample RHC1	-0.08	0.08	.320			0.01	0.07	.913		
Sample RHC2	0.19	0.04	< .001			0.18	0.04	< .001		
Step 2				.11 (.07)	< .001				.09 (.05)	< .001
Sample RHC1	-0.06	0.07	.415			0.00	0.07	.989		
Sample RHC2	0.18	0.04	< .001			0.16	0.04	< .001		
SJ	0.17	0.07	.014			0.25	0.07	< .001		
Atonement HC 1	-0.04	0.07	.545			-0.12	0.07	.106		
Atonement HC 2	-0.22	0.04	< .001			-0.12	0.04	.005		
Step 3				.11 (.00)	.689				.09 (.00)	.487
Sample RHC1	-0.06	0.07	.455			0.00	0.07	.952		
Sample RHC2	0.18	0.04	< .001			0.16	0.04	< .001		
SJ	0.18	0.07	.012			0.25	0.07	< .001		
Atonement HC 1	-0.05	0.07	.537			-0.12	0.07	.104		
Atonement HC 2	-0.21	0.04	< .001			-0.12	0.04	.006		
SJ X Atonement HC 1	0.02	0.08	.817			-0.06	0.08	.436		
SJ X Atonement HC 2	-0.04	0.05	.408			0.04	0.05	.344		

Note. Sample Reverse Helmert Code 1 (RHC1) is coded nationally representative sample 1 = 1, nationally representative sample 2 = -1, and university sample = 0. Sample Reverse Helmert Code 2 (RHC2) is coded nationally representative sample 1 = 1, nationally representative sample 2 = 1, and university sample = -2. SJ refers to Economic System Justification, which was mean-centered. Atonement HC 1 refers to the Helmert Contrast for atonement conditions which compares medium and high levels of atonement. Atonement HC 2 refers to the Helmert Contrast for atonement conditions which compares the low to the average of the medium and high conditions of atonement.

Table S7

Hierarchical Regression Analyzing Likeability of the Victim

Predictor	High Status Perpetrator					Low Status Perpetrator				
	b	*SE*	*p*	R^2 (ΔR^2)	ΔR^2 *Sig.*	*b*	*SE*	*p*	R^2 (ΔR^2)	ΔR^2 *Sig.*
Step 1				.03	.002				.03	.001
Sample RHC1	0.08	0.04	.064			0.07	0.04	.088		
Sample RHC2	-0.08	0.02	.002			-0.08	0.02	.001		
Step 2				.08 (.05)	< .001				.08 (.05)	< .001
Sample RHC1	0.07	0.04	.107			0.08	0.04	.065		
Sample RHC2	-0.07	0.02	.006			-0.07	0.02	.005		
SJ	-0.14	0.04	< .001			-0.14	0.04	< .001		
Atonement HC 1	0.06	0.04	.170			0.11	0.04	.010		
Atonement HC 2	0.08	0.02	.002			0.06	0.02	.018		
Step 3				.08 (.00)	.944				.08 (.00)	.540
Sample RHC1	0.07	0.04	.103			0.08	0.04	.074		
Sample RHC2	-0.07	0.02	.006			-0.07	0.02	< .005		
SJ	-0.14	0.04	< .001			-0.14	0.04	.001		
Atonement HC 1	0.06	0.04	.179			0.11	0.04	.010		
Atonement HC 2	0.08	0.02	.002			0.06	0.02	.020		
SJ X Atonement HC 1	-0.01	0.05	.770			0.02	0.05	.594		
SJ X Atonement HC 2	-0.01	0.03	.861			-0.03	0.03	.319		

Note. Sample Reverse Helmert Code 1 (RHC1) is coded nationally representative sample 1 = 1, nationally representative sample 2 = -1, and university sample = 0. Sample Reverse Helmert Code 2 (RHC2) is coded nationally representative sample 1 = 1, nationally representative sample 2 = 1, and university sample = -2. SJ refers to Economic System Justification, which was mean-centered. Atonement HC 1 refers to the Helmert Contrast for atonement conditions which compares medium and high levels of atonement. Atonement HC 2 refers to the Helmert Contrast for atonement conditions which compares the low to the average of the medium and high conditions of atonement.

Table S8

Hierarchical Regression Analyzing Positive Personality Traits of the Victim

Predictor	High Status Perpetrator					Low Status Perpetrator				
	b	*SE*	*p*	R^2 (ΔR^2)	ΔR^2 *Sig.*	*b*	*SE*	*p*	R^2 (ΔR^2)	ΔR^2 *Sig.*
Step 1				.02	.027				.03	.001
Sample RHC1	-0.06	0.03	.082			-0.03	0.03	0.38		
Sample RHC2	-0.04	0.02	.040			-0.07	0.02	< .001		
Step 2				.09 (.07)	< .001				.09 (.06)	< .001
Sample RHC1	-0.07	0.03	.031			-0.03	0.03	.397		
Sample RHC2	-0.03	0.02	.135			-0.06	0.02	.002		
SJ	-0.16	.03	< .001			-0.16	0.03	< .001		
Atonement HC 1	0.05	.03	.149			0.04	0.03	.222		
Atonement HC 2	0.04	.02	.046			0.01	0.02	.477		
Step 3				.09 (.00)	.671				.10 (.01)	.133
Sample RHC1	-0.07	0.03	.030			-0.03	0.03	.402		
Sample RHC2	-0.03	0.02	.131			-0.06	0.02	.003		
SJ	-0.16	0.03	< .001			-0.15	0.03	< .001		
Atonement HC 1	0.05	0.03	.155			0.04	0.03	.196		
Atonement HC 2	0.04	0.02	.051			0.01	0.02	.469		
SJ X Atonement HC 1	-0.02	0.04	.530			-0.01	0.03	.693		
SJ X Atonement HC 2	0.01	0.02	.531			-0.04	0.02	.051		

Note. Sample Reverse Helmert Code 1 (RHC1) is coded nationally representative sample 1 = 1, nationally representative sample 2 = -1, and university sample = 0. Sample Reverse Helmert Code 2 (RHC2) is coded nationally representative sample 1 = 1, nationally representative sample 2 = 1, and university sample = -2. SJ refers to Economic System Justification, which was mean-centered. Atonement HC 1 refers to the Helmert Contrast for atonement conditions which compares medium and high levels of atonement. Atonement HC 2 refers to the Helmert Contrast for atonement conditions which compares the low to the average of the medium and high conditions of atonement.

Table S9

Hierarchical Regression Analyzing Empathy Toward the Victim

Predictor	High Status Perpetrator					Low Status Perpetrator				
	b	*SE*	*p*	R^2 (ΔR^2)	ΔR^2 *Sig.*	*b*	*SE*	*p*	R^2 (ΔR^2)	ΔR^2 *Sig.*
Step 1				.02	< .001				.04	< .001
Sample RHC1	0.06	0.09	.466			0.08	0.09	.362		
Sample RHC2	-0.16	0.05	.001			-0.22	0.05	< .001		
Step 2				.14 (.12)	< .001				.17 (.13)	< .001
Sample RHC1	0.03	0.08	.729			0.10	0.08	.229		
Sample RHC2	-0.13	0.05	.009			-0.17	0.05	< .001		
SJ	-0.50	0.08	< .001			-0.56	0.08	< .001		
Atonement HC 1	0.16	0.08	.050			0.20	0.08	.014		
Atonement HC 2	0.16	0.05	.001			0.18	0.05	<. 001		
Step 3				.14 (.00)	.792				.18 (.01)	.438
Sample RHC1	0.03	0.08	.690			0.09	0.08	.271		
Sample RHC2	-0.13	0.05	.009			-0.17	0.05	< .001		
SJ	-0.50	0.08	< .001			-0.56	0.08	< .001		
Atonement HC 1	0.16	0.08	.056			0.20	0.08	.015		
Atonement HC 2	0.15	0.05	.002			0.18	0.05	< .001		
SJ X Atonement HC 1	-0.06	0.09	.512			0.11	0.09	.246		
SJ X Atonement HC 2	-0.01	0.06	.842			-0.03	0.05	.544		

Note. Sample Reverse Helmert Code 1 (RHC1) is coded nationally representative sample 1 = 1, nationally representative sample 2 = -1, and university sample = 0. Sample Reverse Helmert Code 2 (RHC2) is coded nationally representative sample 1 = 1, nationally representative sample 2 = 1, and university sample = -2. SJ refers to Economic System Justification, which was mean-centered. Atonement HC 1 refers to the Helmert Contrast for atonement conditions which compares medium and high levels of atonement. Atonement HC 2 refers to the Helmert Contrast for atonement conditions which compares the low to the average of the medium and high conditions of atonement.

Table S10

Hierarchical Regression Analyzing Perceptions of Severity of the Incident

Predictor	High Status Perpetrator					Low Status Perpetrator				
	b	*SE*	*p*	R^2 (ΔR^2)	ΔR^2 *Sig.*	*b*	*SE*	*p*	R^2 (ΔR^2)	ΔR^2 *Sig.*
Step 1				.06	< .001				.08	< .001
Sample RHC1	0.16	0.07	.019			0.09	0.07	.194		
Sample RHC2	-0.19	0.04	< .001			-0.24	0.04	< .001		
Step 2				.23 (.17)	< .001				.26 (.18)	< .001
Sample RHC1	0.13	0.06	.046			0.11	0.06	.086		
Sample RHC2	-0.16	0.04	< .001			-0.19	0.04	< .001		
SJ	-0.46	0.06	< .001			-0.51	0.06	< .001		
Atonement HC 1	0.16	0.06	.067			0.19	0.06	.002		
Atonement HC 2	0.19	0.04	< .001			0.16	0.04	< .001		
Step 3				.23 (.00)	.675				.27(.01)	.105
Sample RHC1	0.13	0.06	.041			0.10	0.06	.110		
Sample RHC2	-0.16	0.04	< .001			-0.20	0.04	< .001		
SJ	-0.46	0.06	< .001			-0.52	0.06	< .001		
Atonement HC 1	0.11	0.06	.077			0.18	0.06	.003		
Atonement HC 2	0.19	0.04	< .001			0.16	0.04	< .001		
SJ X Atonement HC 1	-0.06	0.07	.386			0.11	0.07	.093		
SJ X Atonement HC 2	-0.01	0.04	.847			0.05	0.04	.222		

Note. Sample Reverse Helmert Code 1 (RHC1) is coded nationally representative sample 1 = 1, nationally representative sample 2 = -1, and university sample = 0. Sample Reverse Helmert Code 2 (RHC2) is coded nationally representative sample 1 = 1, nationally representative sample 2 = 1, and university sample = -2. SJ refers to Economic System Justification, which was mean-centered. Atonement HC 1 refers to the Helmert Contrast for atonement conditions which compares medium and high levels of atonement. Atonement HC 2 refers to the Helmert Contrast for atonement conditions which compares the low to the average of the medium and high conditions of atonement.

Table S11

Hierarchical Regression Predicting Perpetrator's Relative Responsibility for the Incident

Predictor	High-Status Perpetrator					Low-Status Perpetrator				
	b	*SE*	*p*	R^2 (ΔR^2)	ΔR^2 *Sig.*	*b*	*SE*	*p*	R^2 (ΔR^2)	ΔR^2 *Sig.*
Step 1				.04	< .001				.02	.006
Sample RHC1	0.18	0.15	.228			-0.07	0.16	.666		
Sample RHC2	-0.33	0.09	< .001			-0.29	0.09	.001		
Step 2				.13 (.09)	< .001				.11 (.09)	< .001
Sample RHC1	0.13	0.14	.369			-0.04	0.15	.782		
Sample RHC2	-0.29	0.08	< .001			-0.23	0.09	.009		
SJ	-0.63	0.14	< .001			-0.70	0.14	< .001		
Atonement HC 1	0.22	0.14	.114			0.25	0.15	.096		
Atonement HC 2	0.38	0.08	< .001			0.36	0.09	< .001		
Step 3				.13 (.00)	.811				.12 (.01)	.537
Sample RHC1	0.12	0.14	.406			-0.05	0.15	.758		
Sample RHC2	-0.30	0.08	< .001			-0.23	0.09	.008		
SJ	-0.63	0.14	< .001			-0.71	0.14	< .001		
Atonement HC 1	0.23	0.14	.106			0.24	0.15	.106		
Atonement HC 2	0.38	0.08	< .001			0.36	0.09	< .001		
SJ x Atonement HC 1	0.09	0.16	.577			0.09	0.16	.585		
SJ x Atonement HC 2	0.03	0.10	.738			0.09	0.10	.344		

Note. Relative responsibility for the sexual assault was assessed on a zero-sum slider scale from 0 (victim's responsibility) to 10 (perpetrator's responsibility). Sample Reverse Helmert Code 1 (RHC1) is coded nationally representative sample 1 = 1, nationally representative sample 2 = -1, and university sample = 0. Sample Reverse Helmert Code 2 (RHC2) is coded nationally representative sample 1 = 1, nationally representative sample 2 = 1, and university sample = -2. SJ refers to Economic System Justification, which was mean-centered. Atonement HC 1 refers to the Helmert Contrast for atonement conditions which compares medium and high levels of atonement. Atonement HC 2 refers to the Helmert Contrast for atonement conditions which compares the low to the average of the medium and high conditions of atonement.
